# Groundwater depletion causing reduction of baseflow triggering Ganges river summer drying

**DOI:** 10.1038/s41598-018-30246-7

**Published:** 2018-08-13

**Authors:** Abhijit Mukherjee, Soumendra Nath Bhanja, Yoshihide Wada

**Affiliations:** 1 Department of Geology and Geophysics, Indian Institute of Technology Kharagpur, Kharagpur, West Bengal India; 2 School of Environmental Science and Engineering, Indian Institute of Technology Kharagpur, Kharagpur, West Bengal India; 3 Present Address: Faculty of Science and Technology, Athabasca University, Edmonton, Alberta Canada; 4 International Institute for Applied Systems Analysis, Laxenburg, Austria; 5 Department of Physical Geography, Utrecht University, Utrecht, Netherlands

## Abstract

In summer (pre-monsoon) of recent years, low water level among the last few decades, has been observed in several lower Indian reaches of the Ganges (or Ganga) river (with estimated river water level depletion rates at the range of −0.5 to −38.1 cm/year between summers of 1999 and 2013 in the studied reaches). Here, we show this Ganges river depletion is related to groundwater baseflow reduction caused by ongoing observed groundwater storage depletion in the adjoining Gangetic aquifers (Ganges basin, −0.30 ± 0.07 cm/year or −2.39 ± 0.56 km^3^/year). Our estimates show, 2016-baseflow amount (~1.0 × 10^6^ m^3^/d) has reduced by ~59%, from the beginning of the irrigation-pumping age of 1970s (2.4 × 10^6^ m^3^/d) in some of the lower reaches. The net Ganges river water reduction could jeopardize domestic water supply, irrigation water requirements, river transport, ecology etc. of densely populated northern Indian plains. River water reduction has direct impact on food production indicating vulnerability to more than 100 million of the population residing in the region. The results of this study could be used to decipher the groundwater-linked river water depletion as well as the regional water security in other densely populated parts of the globe.

## Introduction

**Figure 1:**
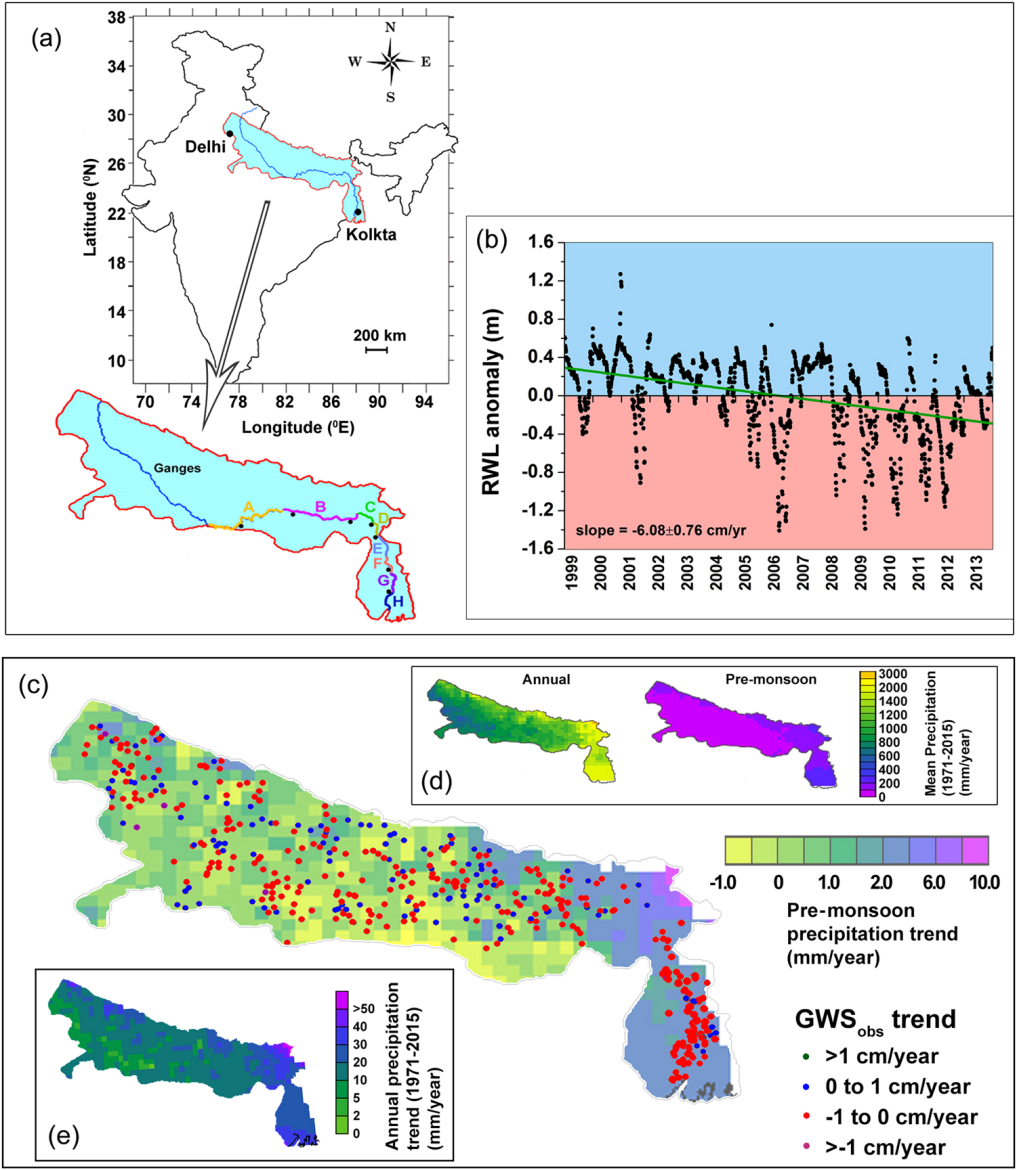
(**a**) Map of the Ganges river (the 8 middle to lower reaches, marked A through H) and adjoining Gangetic alluvial aquifer in parts of the Ganges basin (Mukherjee *et al*.^8^). Major cities are shown; (**b**) Time-series of river water level anomalies (RWLA) at a location in reach F (inset); (**c**) Long-term (1978–2014) trend of change in groundwater storage (GWS_obs_), calculated from *in situ* (marked as solid circles) observations, overlaid on the long-term pre-monsoon precipitation (1971–2015) trend in the area; (**d**) The long-term (1971–2015) mean annual and mean pre-monsoon precipitation is shown in top inset; (**e**) Long-term (1971–2015) trends in total annual precipitation are shown in bottom inset. All the maps were made using Ferret program (NOAA), QGIS software (version 2.12) and standard graphical illustrators.

The Ganges, one of the largest rivers in the world, whose plains have hosted the Indian civilization (Ganges basin in India: ~8.6 × 10^5^ km^2^) for more than the last three thousand years, and presently sustaining one of the largest and densest global populations (i.e. ~10% of global population), has been under increasing stress for last few years in terms of its deteriorating water quality^1^. However, following trends in recent years, the summers of 2015–17 have witnessed an unprecedented low water level and flow in the middle and lower reaches of the Ganges river in India (Reaches A through H, ~1050 km, Figs 1a, S1)^2^. Consequently, surface drinking water plants, power generation units, irrigation systems and navigation along the river have been acutely affected, distressing >120 million downstream residents and other riverine life-forms^3^. Being a pathway of sewerage disposal of much of northern India, the river is now believed to be one of the most polluted mega-rivers of the world^4^. However, the observed summer drying in the recent years, in the studied reaches of the river, pose a much bigger crisis, possibly suggesting an impending surface water crisis in the region, in conjunction with the already well documented, ongoing ‘groundwater drought’ in the Indian subcontinent^5^. Interestingly, the pre-monsoon as well as annual long-term (1971–2015) precipitation trend has been found to be increasing in the study area (Fig. 1b).

The interactions between river water and groundwater are determined by the relative difference between groundwater level and river stage. A river is defined as “gaining” when it is maintained by groundwater seepage (baseflow). It may also be defined as “losing” type if river water infiltrates the adjoining aquifer, or the two-way exchange rivers are controlled by seasonal water levels^6^. The Ganges river has been described as a perennial, gaining river, being sustained by groundwater discharge (as baseflow), specifically during the non-monsoon, dry periods^1,4,7–10^ and with maximum flows in the 4 months (June-September) of monsoon season contributed from increased overland flow (>70% of flow from rainfall)^11^. In general, the river stage have been suggested to be sustained from overland flow from rainfall in basin hinterland, Himalayan glacial melt [~1500 mm/year]^12^, along with discharge from groundwater. Therefore, it is a challenging task to quantify the river water availability over the years in the densely populated region. Climate change scenario^13^ and land-use change as a consequence of population growth^14^ further aggravate the situation. In this context, we have tried to investigate the scenario using a combination of *in situ*, remote sensing based and numerical modelling estimates, along with chemical and isotopic signatures. Our study objective is to answer the following research questions for study domain:

1. What are the rates of dry season, Ganges river water level change over the years?
2. What are the primary, natural influencing factors that are resulting to the observed changes in the lower Indian Gangetic river reaches?
3. How does the rapid groundwater abstraction in the adjoining north Indian Gangetic aquifers might, influence the Ganges river water quantity over years, by altering river water-groundwater interactions?
4. What are the implications of the potential river water depletion to the food security in the region?

## Results and Discussions

Since, the Ganges is a transboundary river, the river water level (stage) and discharge data are geopolitically sensitive and mostly not available in public domain (i.e. classified). Notwithstanding this data unavailability, here, for the first time, we demonstrate (Fig. 1a) the river water depletion trend at the rate of −6.08 ± 0.76 cm/year from *in situ*, daily-scale, long-term (pre-monsoon [summer], 1999–2013) river water level measurements (Reach F, Fig. 1a). We also observed depleting pre-monsoon water level in Ganges river through remote sensing (RS) observation obtained from 28 locations on Ganges river (ENVISAT data^15^, pre-monsoon, 2003–2010), 19 of 28 locations shows decreasing Sen’s slope estimates^16^ of stage ranging from −0.5 to −38.1 cm/year. The remotely sensed runoff data (pre-monsoon, 1998–2017, retrieved from multiple passive microwave radiometry sensors for the 5 locations along the Ganges river^17^) also suggest pre-monsoon depleting trend at rates of −0.07 ± 0.02, −0.07 ± 0.01, and −0.04 ± 0.03 cm/year in reach D, E and F, respectively. Additionally, we have also used Normalized Difference Water Index (NDWI, derived from Aqua MODIS, MYD09A1 product^18^) for observing the pre-monsoon channel width coverage (although, acknowledging that the NDWI can also influenced by sediment load variability in the river). The spatial mean NDWI anomalies (NDWIA) have been decreasing at rate of −0.002 to −0.014/year, particularly at the lower reaches, D through H. Thus, both the *in-situ* and remotely sensed observations suggest that the pre-monsoon Ganges river water is continuously depleting in the studied reaches within the study period (Fig. S8).

**Figure 2:**
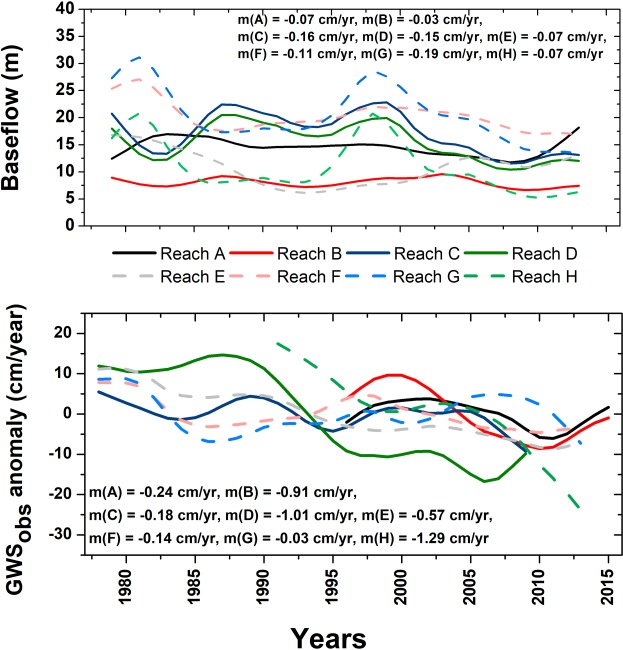
(**a**) Long-term HP trends for simulated pre-monsoon baseflow obtained from global hydrologic model PCR-GLOBWB for the reaches A through H. (**b**) Long-term HP trends for GWS_obs_ anomaly as calculated in reaches A through H.

The mean annual groundwater baseflow contribution to the headwater of the Ganges (in and around Devaprayag in the Himalayas) has been previously estimated to be 48–56%^4^. In absence of data on physical measurements of baseflow^4^, here, we used mixing calculations of stable isotopic (^18^O, ^2^H) composition of groundwater (n = 227) and Ganges river water (n = 22), and groundwater hydrochemistry (n = 560) for hydrogeochemical modelling, sampled by us during the study period from the river and aquifers adjoining the reaches A through H, respectively. The estimation suggests recent baseflow of 14.0% to 26.1% (median 19.4% ± 4.8%) of the present summer water volume of the Ganges in the studied reaches. Simultaneously, we estimated the glacial and snow-melt contribution in our sampled river water to be <5% in all of the studied reaches, consistent with the result obtained by Siderius *et al*.^19^. We also computed baseflow (BF_sim_) for 1979–2013 in a global-scale hydrological model, PCR-GLOBWB^20,21^. HP trends of pre-monsoon BF_sim_ indicate decreasing trend in most (except A and E) of the reaches (Fig. 2a); BF_sim_ depleted at a rate of −1.9 cm/year (−2.6 cm/year, Sen’s slope) in reach B to −28.2 cm/year (−18.7 cm/year, Sen’s slope) in reach G, respectively (Fig. 2a).

Hence, to preserve the river water mass-balance, the observed depletion of Ganges river could suggest decreased inflow from one or more of these aforesaid natural water sources. To understand the influence of overland flow on the observed depletion, we studied long-term trend (1971–2015) of annual and pre-monsoon (dry season) precipitation (4–18% of total annual rainfall, Fig. S4) across the study area in the Gangetic basin, observing an increasing rate in rainfall (Fig. 1b), which is contrary to reduction that could have been expected to the decreased overland flow and thereby Ganges river water depletion (Fig. S3). Moreover, contrary to the present belief of glacial dependency of Ganges river in the dry season, the contribution of glacial runoff and seasonal snowmelt to mean annual flow (~12,000 m^3^/sec river flow at our Reach D)^11^ of entire Ganges basin has been evaluated to be only <10%^11,12,19,22^, and the glacial melt contribution are mostly available in the upper reaches^12^. Hence, with increased precipitation and limited contribution from glacier melt, any major, long-term decrease in the Ganges river water may be largely linked to groundwater-river water interactions, besides any anthropogenic alteration (e.g. river engineering, canal diversion, surface water abstraction and/or related issues and processes; though these were not incorporated in this study due to lack of availability of data).

Groundwater abstraction by distributed pumping in adjoining aquifers could intervene river flow by baseflow reduction or increase in streamflow capture^23^. At the start of pumping regime, the abstracted water is predominantly sourced from the groundwater storage, which, depending on physical characteristics of the aquifer, degree of hydraulic connection between the stream and aquifer, locations and pumping history of wells, could change to adjoining stream water storage over period of days to decades^23^. Such streamflow reduction due to intense groundwater pumping is of utmost concern during low flow seasons^6^ like during pre-monsoon season in Ganges basin. In this study, we found rapid pre-monsoon time depletion of satellite-based groundwater storage (GWS_sat_)^24^ anomaly at a rate of −1.93 ± 0.18 cm/year (−2.09 ± 0.44 cm/year, Sen’s slope) between 2003 and 2015 (trend is significant with *p value* < 0.0001), which matches well with estimates from previous studies^9,25,26^. In this study, we also used continuous *in situ* groundwater level data measured from a network of observation wells for more than last three decades (between 1978–2015, n = 1085) in the adjoining reaches A through H across the study domain, to calculate rapidly declining trends of groundwater storage (GWS_obs_) at a rate of −0.30 ± 0.07 (−0.42 ± 0.07 cm/year, Sen’s slope) cm/year or −2.39 ± 0.56 km^3^/year (−3.39 ± 0.53 cm/year, Sen’s slope) between 1978 and 2015 (trend is significant with *p value* < 0.0001) in the pre-monsoon season. HP trend analyses of GWS_obs_ shows rapid depleting trend in all the reaches, A through H (Fig. 2b); with maximum depletion observed in reaches D [−1.12 ± 0.16 cm/year] (−1.01 ± 0.17 cm/year, Sen’s slope) and H [−1.26 ± 0.29 cm/year] (−1.29 ± 0.21 cm/year, Sen’s slope), respectively (Fig. 2b). We quantified the effects (causality) of these groundwater abstraction on groundwater baseflow contribution to the Ganges river water from the adjoining aquifers of the studied reaches by Granger causality analyses^27^, which indicate GWS_obs_ anomaly significantly (*p value* < 0.05) causes NDWI anomaly at reaches C, F and H. On the contrary, precipitation significantly causes (*p value* < 0.05) NDWI anomaly, only at reach E. The BF_sim_ reduction was also found to be significantly correlated with the GWS_obs_ anomaly [between 1979 and 2013, r = 0.77, significant with *p value* < 0.001].

**Figure 3:**
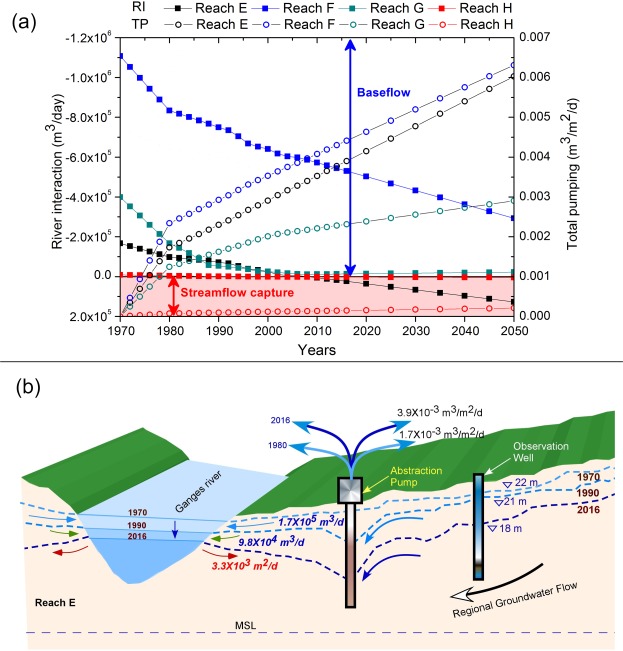
(**a**) Simulated response as generated simulating pre-monsoon river interaction (RI) as baseflow or streamflow capture using a hydrogeologically-detailed regional groundwater flow model^7^ in the adjoining Gangetic alluvial aquifers for the Ganges river reaches E through H for 1970 to present and projected for 2017 to 2050. The observed (1970–2016) and projected groundwater abstraction rates (2017–2050) from these individual reaches are also plotted for comparison. Simulated total pumping values are also shown; (**b**) Conceptual diagram summarizing the hypothetical observations at reach E, indicating Ganges river pre-monsoon stage decrease from 1970–2016.

We also used computed responses of groundwater-Ganges river water interactions in the adjoining aquifers of four lower Indian reaches (E, F, G, H in Fig. 1a; called as Bhagirathi-Hoogly river in these reaches) by numerical simulations (following Cosgrove and Johnson^23^). The simulations were run for river interactions (RI_sim_) for estimating baseflow to the Ganges from the aquifers or streamflow capture from the river, as a consequence of historical^28–31^ and future-predicted groundwater pumping stresses. We used a high-resolution, calibrated, regional groundwater flow model of the lower Gangetic basin of India^7^. It was simulated for pre-monsoon season, for 33 pumping scenarios between 1970 to 2050, e.g. for base-case [*BC*, pre-pumping era i.e. 1970 and earlier], historical pumping condition [*HPC*, 1972–2016], and projected pumping scenarios [*PPS*, based on per-capita groundwater abstraction from 2020 to 2050], with present-day hydrometeorological conditions. Increasing pumping rate has been documented during HPC, and the abstraction trends are expected to increase in future, as suggested in the simulations in all of the four reaches (Fig. 3a). Simulations suggest intricate interactions of Ganges river water with groundwater, characterized by general domination of baseflow from aquifers to the river in recent times (1.0 × 10^6^ m^3^/d in 2016), which (RI_sim_) however, has drastically decreased (by ~59%) in comparison to the RI_sim_ values in 1970 (2.4 × 10^6^ m^3^/d). Computed response curves of potentiometric hydraulic heads (1970–2015, Figs S7–S10) of the aquifers indicate a decline trend of simulated groundwater level and decrease in associated groundwater storage (GWS_sim_) anomaly along reach E (−0.68 ± 0.01 cm/year) (−0.64 ± 0.02 cm/year, Sen’s slope), reach F (−0.42 ± 0.02 cm/year) (−0.35 ± 0.00 cm/year, Sen’s slope), reach G (−0.70 ± 0.05 cm/year) (−0.53 ± 0.03 cm/year, Sen’s slope), and reach H (−0.51 ± 0.07 cm/year) (−0.27 ± 0.04 cm/year, Sen’s slope), respectively (Fig. 3a). The GWS_sim_ anomalies match well with the GWS_obs_ anomalies (1978–2015, n = 540, r = 0.62 to 0.96, significant with *p values* < 0.02) in all the four reaches. The RI_sim_ significantly correlate with GWS_sim_ [r = 0.92 to 1, *p values* < 0.001] and GWS_obs_ [r = 0.62 to 0.93, *p values* < *0*.*02*]. In 1970s (*BC*), all of the four modelled reaches of the Ganges were estimated to be gaining, however, during *HPC*, from *c*. 2010, two of the reaches (E and H) were found to have transformed to losing stream with initiation of stream flow capture (2.6 × 10^4^ m^3^/d in 2016). Further, reach G is in verge of breakthrough from gaining to losing stream in near future (Fig. 3a) with same trend of pumping. With the predicted-future trends of abstraction (*PPS*), the streamflow capture would almost double (>80%) in 2050 (1.3 × 10^3^ m^3^/d) from present, while the baseflow (6.1 × 10^5^ m^3^/d) would decrease further by 38% from present (2016) and 75% of pre-pumping regime of 1970 (Fig. 3a; see b for a paradigm of reach E).

The depletion in river water volume will also have profound effect on future food security in Ganges basin, typically known as the “bread-basket of South Asia”^8^. The highly productive Indo-Gangetic basin would experience substantial reduction in food production, if groundwater is continued to be extracted in current unsustainable rate^32^. We assess that in present times, surface water irrigation for cropping account for 27%^33^ of the total irrigation in the study area. Hence, the dwindling of Ganges river would also severely affect water available for surface water irrigation, with potential future decline in food production. Consequently, according to our estimate, by 2050, the total carbohydrate-based food would be unavailable for almost 1/5^th^ (~115 million) of the >500 million inhabitants of the study area. This suggests a strong need for implementing the adaptation options related to either food production or water use in agriculture in the region e.g. water efficient agricultural practices (e.g. modification of existing flood irrigation techniques), conversion to lower-water consuming crops, reduced groundwater pumping, aquifer rejuvenation and managed recharge (e.g. Bhanja *et al*.^26^) etc.

Hence, excluding human interventions (e.g. river engineering, canal diversion and their related issues etc.), the decline of Ganges water in the lower reaches during recent summers can be related to reduction of groundwater baseflow contributions (BF_sim_ or RI_sim_) to the Ganges river, caused by depletion of groundwater storage (GWS_obs_/GWS_sat_/GWS_sim_) in the adjoining Gangetic aquifers. Our results from groundwater storage estimations from a combination of more than three decades of *in situ* groundwater level measurements, global and regional hydrologic simulations suggest that the dwindling of the baseflow to the gaining reaches of the Ganges river might have started in post-1970s, which eventually might have resulted to transformation of the gaining reaches to losing reaches in recent years. Predictions suggest that the average groundwater contribution to the river can substantially decrease in future. In conjunction to the predicted climate change and dwindling of the Himalayan glacial retreat as suggested by previous study^34^, the future situation can be catastrophic, raising question about the existence of this mega-river that sustains (providing both water and food) the densest and largest riverine population in the world, over the years. Thus a serious appraisal is warranted for the existing policies of pervasive groundwater abstraction from the high-yielding Gangetic aquifers and management methods for sustaining the natural groundwater-Ganges water dynamics that is important for ensuring food security in terms of irrigated agriculture and maintaining ecologic stability requiring environmental flow, specifically in the lower reaches. Proper policy interventions (e.g. managed aquifer recharge with excess precipitation water, channelizing of excess river discharge to interim storage and re-distribution to dryer hinterlands, surface check dams, redistribution of surface water through recharge canals etc.) may result in rejuvenation of the affected aquifers, thereby restoring the natural aquifer hydraulics, as has been recently observed in some of the western, central and southern parts of India^26^.

## Methods

### Remote-sensing estimates of surface water

#### Satellite-based estimates of river water level and flow

The river water level data have been obtained from the European Space Agency (ESA)’s Environmental Satellite (ENVISAT) retrievals between 2002 and 2010 for 28 locations along the Ganges river channel. The radar altimetry information from ENVISAT sensor has been used to obtain the water level and it is matching pretty well with the *in situ* observations^15^. The river runoff/discharge data has been obtained from the archives of the Dartmouth flood observatory’s River Watch Version 3.4. We have used data from the 5 locations distributed across 5 selected reaches. In order to measure the river runoff/discharge, the passive satellite microwave radiometry information through TRMM, AMSR-E, AMSR-2 and GPM sensors are used^17^. The details on the river runoff/discharge measurement methodology can be found in Brakenridge *et al*.^17^.

#### Normalized difference water index (NDWI)

In order to provide information on surface water, we have computed the normalized difference water index (NDWI)^18,35^ using high resolution surface reflectance product from the archives of Moderate Resolution Spectrophotometer (MODIS) along the Ganges river channel (Reaches A through H, extending ~1050 km in the middle and lower courses of the Ganges, Fig. 1a). We used band 2 (near infrared, 841–876 nm)^36^ and 4 (green, 545–565 nm)^36^, from MODIS AQUA 8-day surface reflectance products, MYD09A1 between 2003 and 2016^35^. The same spectral combination has been used in several past studies for detection of surface water bodies^18^ and also for very small water bodies^37^. We used the high resolution (~450 × 450 m) product from the MODIS tiles h25v06 and h26v06. In order to show the surface water extent along the Ganges river channel, we have selected 1252 pixels across the reaches A through H and computed NDWI values for each of the pixels. NDWI anomalies (NDWIA) are calculated after removing all time mean NDWI data from individual NDWI values. Normalized NDWI anomalies are calculated after dividing temporal standard deviation from its individual values. Although the surface water level anomaly (SWLA) calculated using measured river water level, which is not directly equivalent to the NDWIA, however, NDWI data would provide near-accurate surface water information^38^ in areas with restricted or no available measurements. While comparing with ground-based river water level estimates, we found good match of NDWIA in two locations within reaches A and F (Fig. S5a and b).

### Groundwater level measurements

Water level data were obtained from Central Ground Water Board (CGWB, India) for measurements between January 1996 and November 2015 in areas up to reach B. Water level data were measured from the first available aquifer at a total number of more than 2000 wells with temporal resolution of four times a year (late post-monsoon [January], pre-monsoon [May], monsoon [August] and early post-monsoon [November])^39^. We also used groundwater level data for downstream locations, reaches C through F from the Government of West Bengal, at more than 500 wells between 1978 and 2013. Groundwater level anomalies (∆h) are estimated by subtracting groundwater level depth from its all time period mean and following reversing the sign. Observed GWS (GWS_obs_) were obtained by multiplying specific yield (S_y_) with ∆h values. The reported data values contain linear regression analysis results and the Sen’s slope estimates are also given within brackets. The errors associated with linear regression and Sen’s slope estimates are provided for one-sigma trend error for the estimation. S_y_ values ranged between 0.06 and 0.20 within the study area^24^. We used a specific yield value of 0.13 (median value of 0.06 and 0.20) in the entire Ganges basin following Bhanja *et al*.^24^. Observed GWS anomalies are obtained after removing all time mean GWS from individual GWS data.

### Gravity Recovery and Climate Experiment (GRACE)

133 monthly GRACE RL05M mascon solutions are obtained from the Jet Propulsion Laboratory, National Aeronautics and Space Administration (NASA)^24,40^ between January 2003 and December 2014, in order to determine terrestrial water storage (TWS) anomaly. Groundwater storage (GWS_sat_) anomalies are computed by removing soil moisture (SM), and surface water (SW) equivalents from TWS anomalies. SM and SW equivalents are obtained from the archives of Global Land Data Assimilation System (GLDAS)^41^. Outputs from a combination of three different models’, Community Land Model (CLM2), Variable Infiltration Capacity (VIC), and NOAH are used here. The CLM simulates soil moisture in 10 different layers (0 to 343 cm) and the other two models, VIC and NOAH simulates in 3 layers (0 to 190 cm) and 4 layers (0 to 200 cm), respectively. In order to determine anomaly for any of the component, all time mean value for that component has been removed from each individual data of that component. For comparison with observed GWS anomalies, GRACE based estimates are tuned to the observational time period only i.e. four times a year between 2003 and 2014. We used 0.25° × 0.25° daily gridded precipitation data from India Meteorological Department (IMD) data^42^ total precipitation product between 1970 and 2014. The errors associated with linear regression and Sen’s slope estimates are provided for one-sigma trend error for the estimation.

### Surface water data

NDWI has been previously successfully applied in several studies for characterizing open water bodies^38,43^. We validated the NDWI data (2003–2015) for the Ganges, with *in-situ*, temporal river water level data collected at two locations in Reaches A and H (daily data, with maximum range 1999–2016), with a statistically significant level at 0.1. Based on the observed NDWI values, the years are categorized in all of the locations (Fig. S8), also indicating occurrence of comparatively higher rank values (i.e. lower NDWI occurrence) in lower reaches (location number 700 onward) in recent years (Fig. S8).

### Groundwater modelling

The groundwater flow modelling was done by simulating 33 different pumping scenarios under observed climate condition and without any climate change forcing in an updated version of the robust, calibrated model groundwater flow of the ~21,000 km^2^ Gangetic aquifers in the Western Bengal basin, as reported in Mukherjee *et al*.^7^. A block-centric, finite-difference grid was used for dynamic-equilibrium simulations in MODFLOW^44^. Details are provided in supplementary information.

### Isotope and Hydrogeochemical analyses

In order to find out the percentage contribution of groundwater in Ganges river water, a three component isotope mixing model has been used to characterize the fractions of glacial/ice melt (ICE), surface runoff (SR) and the groundwater discharge/baseflow (GW) component. It is assumed that the total volume of river water is composed of three major components: glacial/ice melt water from upper reaches, surface runoff components and groundwater discharge/baseflow. A three component mixing model has been used to separate the fractions of these three components from the total water. Details can be found in Supplementary information.

Geochemical analysis are used to characterize of geochemical pathways and processes that accounts for the chemical changes leading to hydrogeochemical evolution of the shallow groundwater because of mixing with inflowing Ganges river water reaches from the vicinity. Ion activities and saturation indices were calculated using PHREEQC^45^ version 2.17. The groundwater flow system and chemical composition of the aquifer are assumed to be at steady-state conditions. A total of 76 different inverse geochemical models in the eight aquifers were executed within the acceptable uncertainty range, resulting to 1377 solutions. Detailed descriptions are given in Supplementary Information.

### Assumptions and uncertainty analyses

Apart from the assumptions provided in the individual sections, i.e. isotope analysis, food security estimates etc., we have reached the conclusion based on our analysis on available dataset. Information on human impact on the Ganges river, including river engineering, canal diversion, evapotranspiration etc. were not available and hence, not included in any of the estimations. Unavailability of all of the data for the entire time period restrict us to compare the individual data for the available period only. Future projections are computed based on the present scenario.

We have estimated one-sigma trend error for linear regression analysis in the GRACE TWS anomaly estimates (σ_TWS_). In order to calculate the errors associated with soil moisture anomaly (σ_SM_) and surface water anomalies (σ_SR_), standard deviations of the trends of the three GLDAS models are determined. Subsequently, error estimates in the trend of GWS anomaly (σ_GWS_) are estimated following the equation,

1 $${\sigma }_{{\rm{GWS}}}=\sqrt{[{({{\rm{\sigma }}}_{{\rm{TWS}}})}^{2}+{({{\rm{\sigma }}}_{{\rm{SM}}})}^{2}+{({{\rm{\sigma }}}_{{\rm{SR}}})}^{2}]}$$

Errors in linear regression estimates for GWSA_obs_ and other data were obtained by estimating one-sigma trend error for calculating the linear trend.

The uncertainty associated with the calculation of mean baseflow value through three component isotope analysis is presented as the 1 standard deviation from the mean value^46,47^. The uncertainty associated with river water discharge values are represented as the 1-sigma deviation from the mean values.

## Electronic supplementary material

Supplementary Information
