# Supplementary material for: Groundwater depletion causing reduction of baseflow triggering Ganges river summer drying
Source: Sci Rep. 2018 Aug 13;8:12049. doi: 10.1038/s41598-018-30246-7 (PMC6089894; doi:10.1038/s41598-018-30246-7)
Supplement: Supplementary file 1 — Supplementary Information [file 41598_2018_30246_MOESM1_ESM.pdf]

Groundwater depletion causing reduction of baseflow triggering Ganges river summer drying

Abhijit Mukherjee[1,2 *], Soumendra Nath Bhanja[1,3] , Yoshihide Wada[4,5]

[1]Department of Geology and Geophysics, Indian Institute of Technology Kharagpur, WB, India

[2]School of Environmental Science and Engineering, Indian Institute of Technology Kharagpur, West Bengal 721302, India.

[3]Presently at Faculty of Science and Technology, Athabasca University, Alberta T9S3A3, Canada.

[4]International Institute for Applied Systems Analysis, Laxenburg, Austria.

[5]Department of Physical Geography, Utrecht University, Utrecht, Netherlands.

Abhijit Mukherjee and Soumendra Nath Bhanja contributed equally to this work.

Contents of Supplementary Information

S1 Study locations

S2 Gravity Recovery and Climate Experiment (GRACE) calculations

S3 Baseflow simulation using PCR-GLOBWB

S4 River reach response analyses by groundwater flow modeling

S5 Hydrogeochemical and isotope modeling

S6 Non-parametric statistical analysis

S7 Food security estimates

S1 Study locations

We have selected eight major Ganges river reaches in its middle and lower course and their adjoining aquifers in India, in which various hydrogeological and hydrogeochemical parameters were sampled and/or measured. The river water is sampled at 22 locations (Fig. S1). Groundwater samples (n=227 for stable isotope analysis; n=560 for hydrochemical reaction modelling) are collected from the aquifer locations adjoining the river channel.

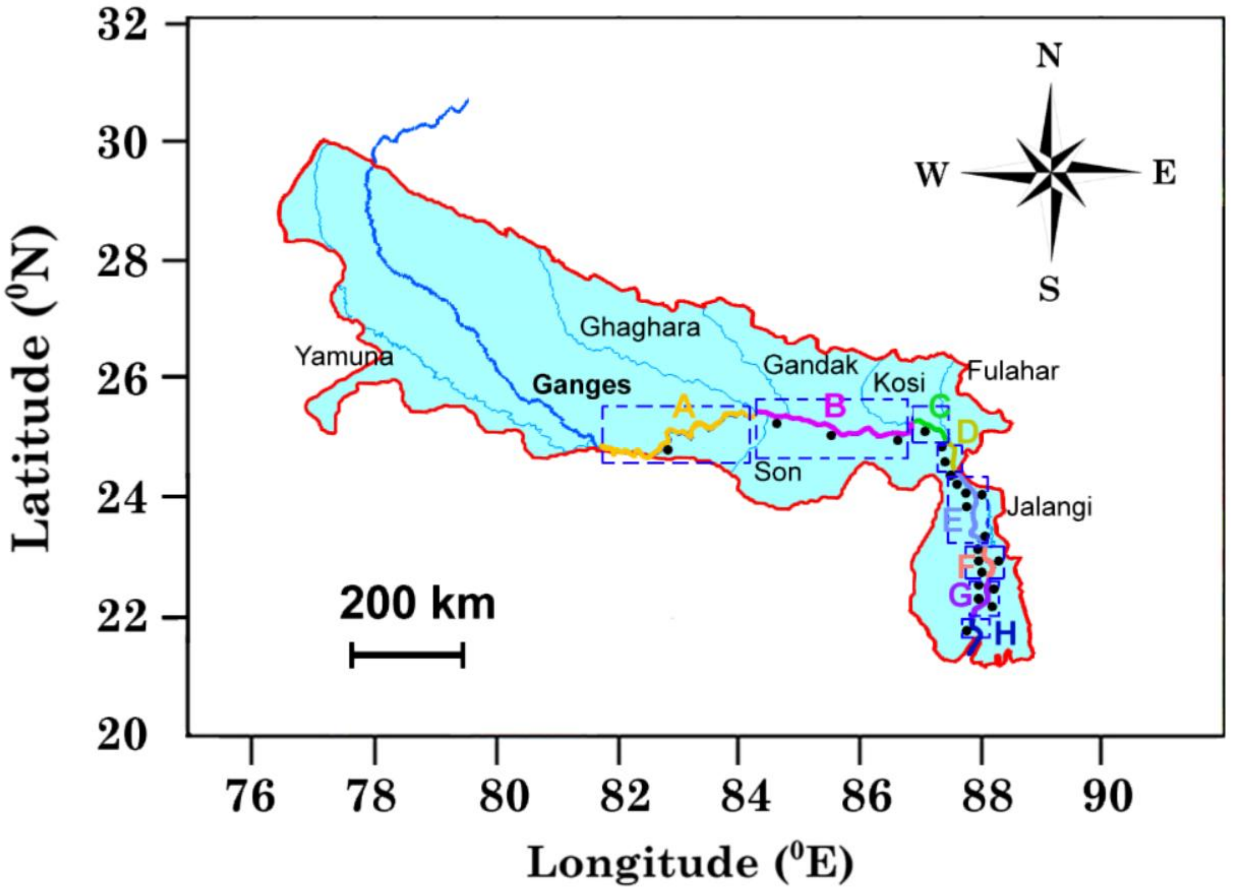

Fig. S1: The Ganges river and its major tributaries. The reaches are shown in different colors. The black filled circles are representing the locations for river water sampling (n=22). The groundwater sampling locations are shown through dashed rectangles. The reaches are selected based on the confluence points of major tributaries. All the maps were made using QGIS software (version 2.12) and standard graphical illustrators

S2 Gravity Recovery and Climate Experiment (GRACE) calculations

The mascon solutions are derived from the change in distance between two GRACE satellites between January 2003 and December 2014. Recently released RL05 mascon solutions are obtained from the Jet Propulsion Laboratory, National Aeronautics and Space Administration (NASA) in order to get terrestrial water storage (TWS) change information (Watkins et al., 2015; Bhanja et al., 2016). Several techniques are applied on the data for obtaining TWS solutions (http://grace.jpl.nasa.gov/data/get-data/jpl_global_mascons/ accessed on 26 April, 2016). Degree 2 and order 0 coefficients in the data are replaced by the coefficients derived from Satellite Laser Ranging reported by Cheng *et al.* (2011). Degree 1 coefficients are derived from a process described in Swenson *et al.* (2008). Post glacial rebound signal in the data are removed by the process developed by Geruo and Wahr (2013). The entire globe is divided into 3^0 equal area spherical mass concentration blocks in the mascon approach (Watkins et al., 2015). Introduction of the a priori information lead to removal of correlated noise (stripes), therefore, post-

processing filters are not required to apply on the data- this is the major improvement from the spherical harmonics approach in terms of reducing inherent errors (see the details in Watkins *et al.,* 2015).

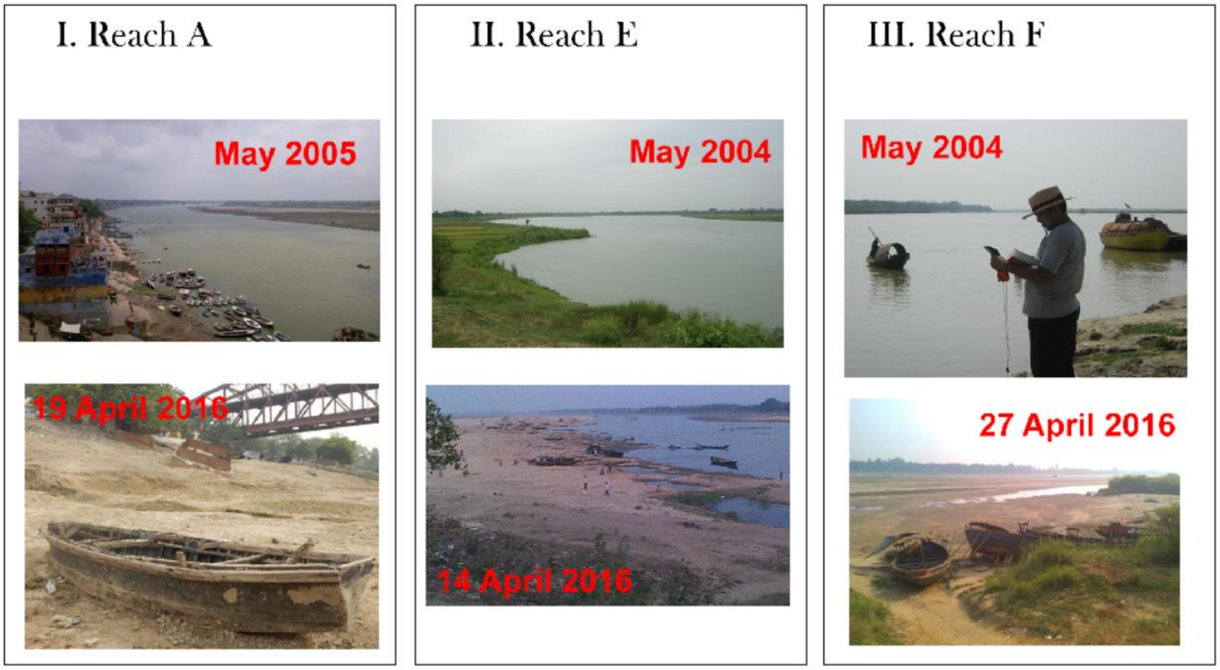

Fig. S2: Photographs of the Ganges river channel water in reaches A, E and F taken by the authors in pre-monsoon (May) 2004-2006 and marked dates in 2016, to demonstrate the discernable, aggravated, ongoing summer drying of the river. The locations of the photographs might not be exactly same, they are used for indicative purpose only

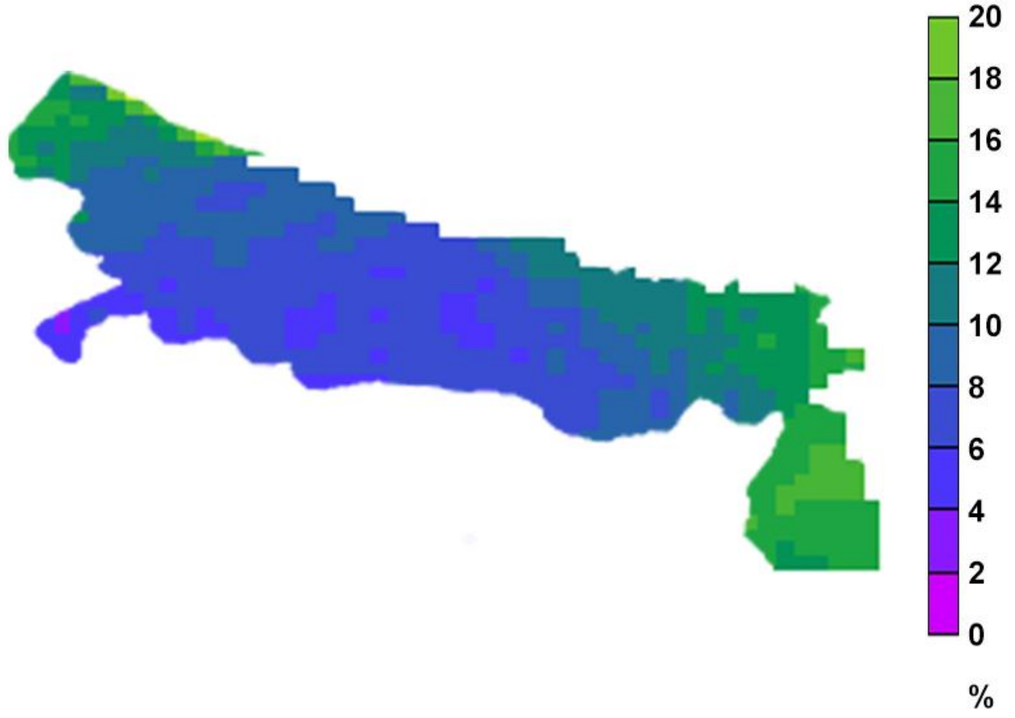

Fig. S3: Percentage ratio of gridded ($0.25^0 \times 0.25^0$) premonsoon-time precipitation to total annual precipitation in 1971-2015. All the maps were made using Ferret program (NOAA) and standard graphical illustrators

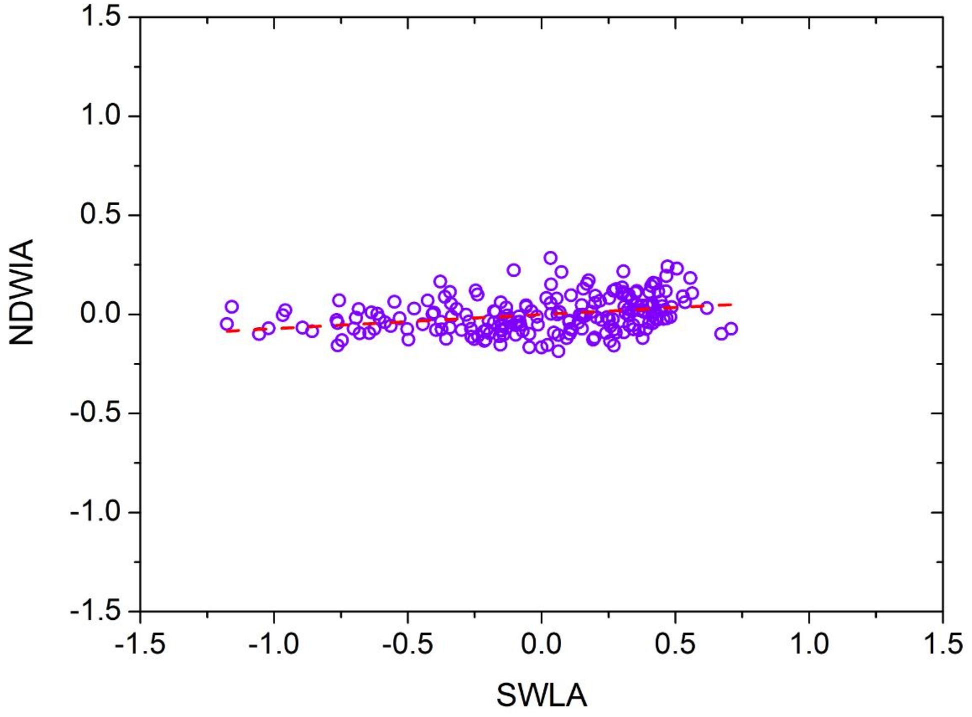

.

Fig. S4a: NDWIA and surface water level anomaly at a location within reach F. The data are shown in premonsoon time 8-day interval between 2003 and 2013

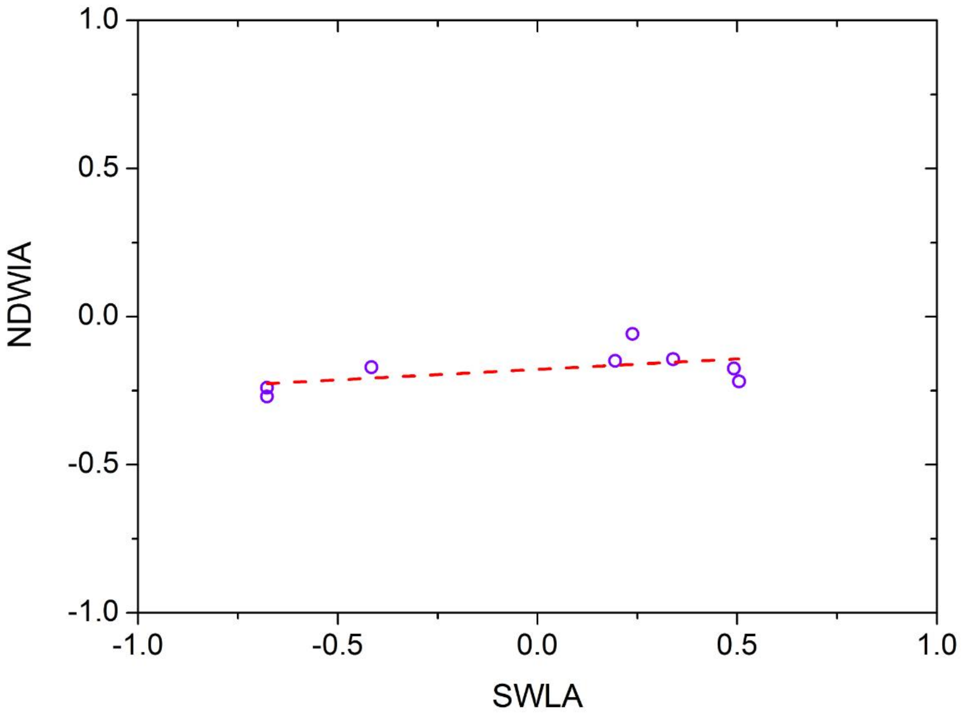

Fig. S4b: NDWIA and surface water level anomaly at a location within reach A. The data are shown in premonsoon time 8-day interval in 2016.

S3 Baseflow simulation using PCR-GLOBWB

The global-scale hydrological model, PCR-GLOBWB, have been used to simulate baseflow at a resolution of $0.1^0 \times 0.1^0$ (Wada et al., 2014). The PCR-GLOBWB model has three soil layers (Fig. S5), the first two layers are not directly connected to groundwater. The third and deepest soil layer in PCR-GLOBWB model is considered to be a groundwater reservoir replenished by active recharge from the upper layers (Wada et al., 2014). Groundwater recharge is calculated after removing capillary rise from the deep percolation. The baseflow (Q_b) is characterized as drainable water from the reservoir to the river. A first order linear reservoir approach has been implemented in the model to compute groundwater storage and baseflow (Wada et al., 2014). In the model, baseflow is a product of groundwater storage (GW_{S3}) and recession coefficient (Wada et al., 2014). The detailed methodology can be found in Wada et al. (2014). Recession coefficient, J, represents average residence time of groundwater. Global reservoir coefficient values are computed by combining global drainage length and lithology information (Wada et al., 2014). Finally, local runoff is computed as a sum of direct runoff, interflow and baseflow. The groundwater layer of PCR-GLOBWB has been represented using a 1-layer MODFLOW model (Wada et al., 2016). The MODFLOW model is infused with groundwater recharge and the river discharge data from the PCR-GLOBWB data (Wada et al., 2016). River and drain package of MODFLOW has been used to model the groundwater and surface water interaction (Wada et al., 2016). Three different types of rivers and streams are considered to compute the river discharge: large rivers (width >25 m), small rivers (width <25 m), and the springs and streams (Wada et al., 2016). The following equations are solved to simulate baseflow in different time period (Wada et al., 2014):

$$GW_{S3,t} = GW_{S3,t-1} - Q_{b,t-1} + (q_{p,S2\rightarrow S3,t} - C_{r,S3\rightarrow S2,t}) \quad (1)$$

$$Q_{b,t} = GW_{S3,t} \times J \quad (2)$$

where, $k_{sat,S3}$ represent the hydraulic conductivity of the saturated layer or aquifer (S3); f_d represents the drainable porosity; aquifer depth is represented by D_c; drainage length is represented as B_c. B_c is estimated through drainage density analyses. The drainable porosity and saturated hydraulic conductivity are linked to a simplified lithological map of the world by Durr et al. (2005). A global map of the reservoir coefficient have been obtained after solving the equation:

$$J = \frac{\pi^2 \, (k_{sat,S3} \times D_c)}{4 \times f_d \times B_c^2} \quad (3)$$

Parameter sensitivity analyses of D_c and $k_{sat,S3}$ have been provided in Sutanudjaja et al. (2011). Local runoff (Q_l) has been estimated as the combination of the direct runoff (Q_d), baseflow (Q_b) and the interflow (Q_i).

$$Q_l = Q_d + Q_b + Q_i \quad (4)$$

River network has been used to route the local runoff.

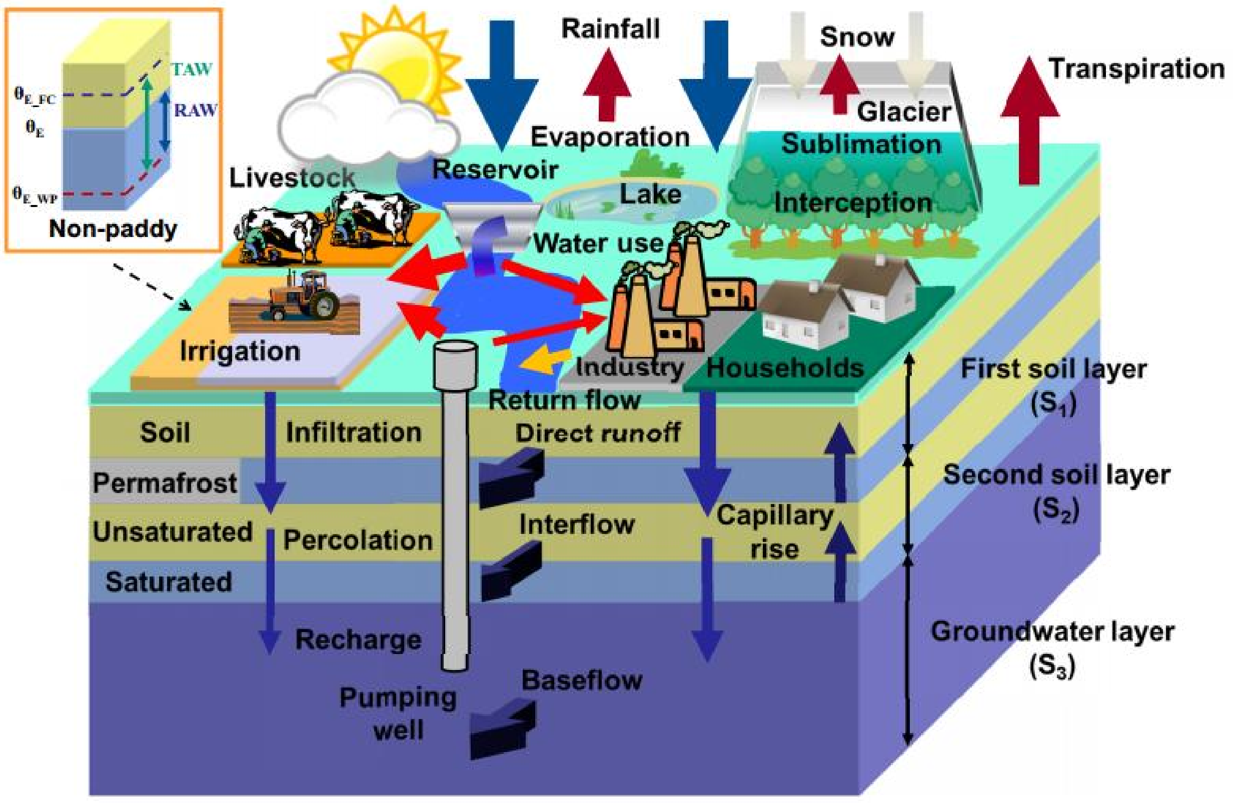

Fig. S5: Schematic diagram of PCR-GLOBWB operation

S4 River reach response analyses by groundwater flow modeling

Discretization: The model domain was discretized at a grid resolution of 1000 m (x) × 1000 m (y) × 15 m (z) for 22 layers from land surface to a depth of 300 m below mean sea level. SRTM 90 DEM with horizontal resolution of 3 arc-sec/90 m; vertical resolution of 1 m was used for surface elevation. Spatial location data were projected to Universal Transverse Mercator (UTM 1983), Zone 45 (central meridian 87, spheroid GRS 80, reference latitude 0, scale factor of 0.9996, false easting 500,000, false northing 0). The coordinates of origin of the modeled area for both type of modeling were easting 600,000, northing 2,380,000. The modeled study area is bounded in the north, west, and east by the Rivers Ganges, Bhagirathi-Hoogly and Jalangi/Ichamati, respectively, and to the south by the Bay of Bengal. The study area is 335 km in length and 110 km in width.

Recharge: The total recharge inflow in the study area was calculated from monthly climatic data obtained from 25 observations stations in and near the study (1811 to 2013) obtained from the NCDC database (http://www.ncdc.noaa.gov/oa/pub/data/ghcn/v2/ghcnftp.html, accessed on January 20, 2015). Based on these data, potential evapotranspiration (PET) values were estimated for each location according to Malmstrom (1969):

$$PET(mm/month) = 40.9 \times e^*(T) \quad (5)$$

where

$$e^* = 0.611 \times \exp\left(\frac{17.3 \times T}{T + 237.3}\right) \quad (6)$$

T is average monthly temperature (°C).

Evapotranspiration (ET) values were calculated (Pike 1964) for each of these 25 locations as:

$$ET(mm/month) = \frac{P}{[1 + (\frac{P}{PET})^2]^{1/2}} \quad (7)$$

where, P = average precipitation (mm/month)

PET = potential evapotranspiration (mm/month)

Potential recharge (R_g) was then calculated as:

R_g (mm/month) = P – ET (8)

The monthly R_g values were aggregated according to the previously described seasons. These values were then used to form 2-D daily R_g grids of the study area by kriging in Surfer, where

$$\text{daily Rg (mm/day)} = \frac{R_s}{d_s} \quad (9)$$

where

R_s = cumulative mean monthly rainfall for that season (in mm)

d_s = number of days in that season

Boundary conditions and surface water: Boundary conditions were assigned for head-dependent flux boundaries as specified head for the rivers in the area e.g. Bhagirathi Hoogly (Ganges river), Ichamati river, Churni river, Matla river etc.. Constant-head for the Bay of Bengal was used following the natural physiographic and hydrologic conditions existing in the area, with a buffer $\geq$~20 km on all sides from the domain boundary. The groundwater-river water interaction was simulated by the river package (RIV). Scarcity of surface water data limited invocation of more sophisticated routines. The rivers were incorporated as specified head boundaries, divided into 11 reaches (1 reach for Ganges towards Bangladesh and 4 reaches for the Bhagirathi-Hoogly channel of the Ganges) and were assigned attributes like channel width (W) and length (L), stream stage, channel bed elevation and thickness (T), and sediment type based on data collected from the field or from various sources. Conductance (C) data of river beds for the study area, C values were calculated as (in m^2/day):

$$C = (\frac{K \times W \times L}{T}) \tag{10}$$

where K is the hydraulic conductivity of the bed sediments. The Bay of Bengal was set as a constant-head (0 m) boundary, which was assigned to the southernmost rows at the edge of the study area along the coastline (low tide line).

Hydraulic Parameterization: The hydraulic heterogeneity was incorporated from a fully-developed three-dimensional, finite-difference grid, block-centric lithologic model. Based on the acquired lithologs (269 logs of existing drinking water, irrigation and observation wells), a lithologic model was developed. The lithologic solid model was created using a block-centric finite-difference grid. The solid model was developed to a depth of 300 m below MSL and the topographic elevation was defined by the SRTM 90-DEM grid. The gridding was done by eight nearest-neighbor methodology with 3-D interpolation by average minimum distance. The lithologies in the logs were grouped into three hydraulic types: gravel or sand (aquifer), sandy clay (low hydraulic conductivity aquifer) and clay (aquitard). The sensitivity of the model was tested by varying the horizontal and vertical spacing of the nodes and an optimized final model was constructed that was least sensitive to changes in spatial resolution (i.e., smaller grid sizes). The resolution of the final model was 1000 m (x) × 1000 m (y) × 2 m (z). The resulting discretization consisted of 135 x nodes × 335 y nodes × 165 z nodes, thus having 7,462,125 solid model nodes, each with a voxel volume of 2,000,000 m^3. Four nodes were assigned around each interpolated datapoint. Transmissivity (T) values of sediments were calculated iteratively, using the method of Mace (2001), from 132 specific capacity tests in the study:

$$T_f = \frac{S_c}{4\pi} \ln(\frac{2.25 \times T_i \times t_p}{r_w^{\ 2}\ S}) \tag{11}$$

In this method, T_i is the initially assumed and T_f is the final T value, S_c is specific capacity, t_p is duration of pumping, r_w is the well radius, and S is storativity (set to an average of 0.03[46]). The average hydraulic conductivity (K_x) was calculated from the T and modeled aquifer thickness for the four districts, and K_z assumed equal to 1/10 K_x. Initial Porosity was assigned as 0.2. The final values of the aquifer parameters were ascertained by inverse modeling and model response to sensitivity analyses. The layers were allowed to have seepage from the top and leakage through the base, making them hydraulically connected. The top-most layer was defined

as unconfined and the second layer as unconfined (T varying). The rest of the layers were defined as confined, as the water table was not expected to fall >30 m.

Table S1: Results of sensitivity analyses on PREM-p for hydraulic parameter values.

**Aquifer (K_x) m/d**	**RSS**	**Comment**
25	78.9	formation of large dry cells
37.5	34.4	used model parameter value
50	21.9	formation of small dry cells in upper and middle reaches
70	21.7	formation of large dry cells all over the study area
Aquifer (K_y) m/d		
25	34.4	used model parameter value
37.5	31.2	formation of dry cells near southern reaches
15	39.5	formation of dry cells near northern and southern reaches
Aquitard ($K_{x\text{-}y}$) m/d		
0.1	34.4	used model parameter value
0.5	47.7	not much effect, long time to converge
1	49.1	not much effect, long time to converge
0.01	42.2	distorted contours and flow toward River Ganges
S		
0.03	34.4	used model parameter value
0.015	34.4	no effect
0.06	34.4	no effect
0.3	34.4	no effect
n		
0.2	34.4	used model parameter value
0.1	34.4	no effect
0.4	34.4	no effect

Abstraction and simulation: Groundwater abstraction was simulated from shallow wells (STW) with low to medium yield (10-20 m^3/hr) and high-yield (up to 200 m^3/hr), deep irrigation tube wells (DTW) by electric (18-20 h.p.) turbine or submersible pumps. Their current estimated numbers for each reach for each year till 2007 was obtained from MoWR (1993, 2001, 2005,

2013). The pump numbers for recent years were obtained from informal information. The pumpage for each model grid cell was calculated by following the equation:

$$Q = (\frac{N \times r \times t}{A}) \times a \tag{12}$$

Here, Q = the assigned total pumpage (outflow) value to each grid cell for a specified time (L^3); A = area of each district within the model boundary (L^2); N = number of pumps; r = pumpage rate (L^3/T); t = hours of operation per day (T); a = area of each grid cell. Assuming the rates of population growth similar to growth rates between 1991-2011, projected number of pumps and pumping rates for 2017-2050 for each reach were calculated according to the following equation:

$$N = \eta \times P \tag{13}$$

Where N= projected number of pumps; η = number of pumps per capita in 2011. Response to adjoining river reaches were calculated follow Cosgrove and Johnson

$$Q = \sum_{j=1}^{n} \frac{L_j}{R_j} \tag{14}$$

where L_j is rate of depletion of the river reach j and Rj is the response function.

Inverse modeling was performed for parameter estimation for hydraulic properties and recharge using recent 197 hydraulic-head values. In the inverse mode, initial values of aquifer parameters were based on the values obtained from previous studies or field measurements. The values obtained at the end of the analyses were used for forward simulations and determining the calibration statistics. Sensitivity of hydraulic heads in target wells to model parameters were also analyzed by sequentially varying the hydraulic properties, recharge, stream bed conductance, stages of major streams, and groundwater abstraction.

Calibration and sensitivity analyses: Because water level data were only available for the study area for pre-monsoon in presence of pumping, the PREM-p model was calibrated in steady state against known head values from head values observered from more than 120 locations across the study area. Sensitivity analyses were performed on this calibrated model (PREM-p) by sequentially varying the K_{x-y} of aquifers and aquitards, S and n values. The model was found to be most sensitive to the K_x values of dominant lithologies. Using the selected values from the analyses, the final model was recalibrated and found to have good agreement with present water level and hydraulic-head contours. The differences between the observed and computed values were probably a cumulative manifestation of a specified head boundary in the vicinity, discrepancy in topographic elevations, exclusion of STW, and uncertain aquifer parameters. The finalized values obtained from the sensitivity analyses were then used for defining the aquifer properties of the scenario simulations

Table S2: Results of model calibration of base model with the best-fitted parameter values from table 1.

**Latitude**	**Longitude**	**Layer**	**Observed (hydraulic head in m above MSL)**	**Computed (hydraulic head in m above MSL)**	**Residual**

24.5	88.08	4	22	21.32	0.68
24.42	88.25	4	20.7	20.40	0.30
24.34	88.32	4	19.9	18.38	1.52
24.25	88.6	4	16.5	15.27	1.23
24.23	88.27	4	17.2	18.55	-1.35
24.18	88.27	4	18.6	17.52	1.08
24.15	88.47	4	14.5	11.75	2.75
24.09	88.26	4	17.5	16.34	1.16
23.91	88.46	4	14.3	14.09	0.21
23.86	88.52	6	13.5	13.36	0.14
23.69	88.33	6	11.3	11.05	0.25
23.61	88.56	6	11.9	11.03	0.87
23.6	88.35	6	12.4	10.98	1.42
23.33	88.66	6	8.2	8.24	-0.04
23.05	88.83	7	7.1	6.75	0.35
22.77	88.53	10	4.5	3.64	0.86
22.76	88.79	7	7.8	7.19	0.61
22.73	88.38	9	3	1.77	1.23
22.61	88.68	12	4	6.50	-2.50
22.56	88.42	9	0	-0.18	0.18
22.45	88.43	12	5	4.19	0.81
22.4	88.48	12	5	3.43	1.57
22.3	88.56	12	3.5	2.74	0.76
22.3	88.74	12	2.8	3.04	-0.24
22.16	88.29	12	2.2	1.38	0.82
22.06	88.39	12	2.2	0.89	1.31

Residual mean: 0.61

Residual standard deviation: 0.97

Sum of squares: 34.35

Absolute residual mean: 0.93

Minimum residual: -2.50

Maximum residual: 2.75

Table S3: Summary of groundwater abstraction and river response in terms of baseflow or streamflow capture in the Ganges river reaches D to H

	Abstraction rates ($m^3/m^2/d$)						River Response (m3/d; '+'baseflow/'-' streamflow capture)			
**Year**	**Reach E**	**Reach F**	**Reach G**	**Reach H**	**Reach H (CCU)**	**Total**	**Reach E**	**Reach F**	**Reach G**	**Reach H**
1970	0.0E+00	0.0E+00	0.0E+00	0.0E+00	0.0E+00	0.0E+00	-1.7E+05	-1.1E+06	-4.0E+05	-9.0E+03
1972	3.5E-04	4.7E-04	2.5E-04	1.5E-05	1.8E-04	1.3E-03	-1.5E+05	-1.1E+06	-3.5E+05	-8.0E+03
1974	6.9E-04	9.4E-04	5.0E-04	3.0E-05	3.5E-04	2.5E-03	-1.4E+05	-1.0E+06	-3.1E+05	-6.9E+03
1976	1.0E-03	1.4E-03	7.5E-04	4.5E-05	5.3E-04	3.8E-03	-1.3E+05	-9.4E+05	-2.6E+05	-5.7E+03
1978	1.4E-03	1.9E-03	1.0E-03	6.0E-05	7.1E-04	5.0E-03	-1.1E+05	-8.9E+05	-2.1E+05	-4.7E+03
1980	1.7E-03	2.3E-03	1.2E-03	7.4E-05	8.8E-04	6.3E-03	-9.8E+04	-8.3E+05	-1.7E+05	-3.7E+03
1982	1.8E-03	2.5E-03	1.3E-03	7.8E-05	9.8E-04	6.7E-03	-9.3E+04	-8.2E+05	-1.4E+05	-3.1E+03
1984	2.0E-03	2.6E-03	1.4E-03	8.2E-05	1.1E-03	7.1E-03	-8.9E+04	-8.0E+05	-1.2E+05	-2.7E+03
1986	2.1E-03	2.7E-03	1.5E-03	8.7E-05	1.2E-03	7.5E-03	-8.4E+04	-7.8E+05	-8.9E+04	-2.1E+03
1988	2.2E-03	2.8E-03	1.5E-03	9.1E-05	1.3E-03	8.0E-03	-8.0E+04	-7.7E+05	-5.7E+04	-1.6E+03
1990	2.3E-03	2.9E-03	1.6E-03	9.4E-05	1.3E-03	8.3E-03	-7.2E+04	-7.5E+05	-5.4E+04	-1.5E+03
1992	2.4E-03	3.0E-03	1.7E-03	9.9E-05	1.4E-03	8.6E-03	-6.7E+04	-7.3E+05	-4.9E+04	-1.3E+03
1994	2.5E-03	3.2E-03	1.8E-03	1.0E-04	1.4E-03	9.0E-03	-5.3E+04	-7.0E+05	-4.4E+04	-4.7E+02
1996	2.7E-03	3.3E-03	1.9E-03	1.1E-04	1.4E-03	9.3E-03	-3.9E+04	-6.7E+05	-3.7E+04	5.3E+02
1998	2.8E-03	3.4E-03	1.9E-03	1.1E-04	1.4E-03	9.7E-03	-3.3E+04	-6.5E+05	-2.7E+04	9.6E+02
2000	2.9E-03	3.5E-03	2.0E-03	1.2E-04	1.4E-03	1.0E-02	-2.6E+04	-6.4E+05	-2.6E+04	1.3E+03
2002	3.0E-03	3.6E-03	2.1E-03	1.2E-04	1.4E-03	1.0E-02	-1.0E+04	-6.2E+05	-2.1E+04	1.3E+03
2004	3.2E-03	3.8E-03	2.1E-03	1.3E-04	1.4E-03	1.1E-02	-6.6E+03	-6.0E+05	-1.7E+04	1.7E+03
2006	3.3E-03	3.9E-03	2.1E-03	1.3E-04	1.4E-03	1.1E-02	-5.2E+03	-6.0E+05	-1.3E+04	1.8E+03
2008	3.4E-03	4.0E-03	2.2E-03	1.3E-04	1.4E-03	1.1E-02	-6.8E+02	-5.9E+05	-1.2E+04	1.9E+03
2010	3.5E-03	4.1E-03	2.2E-03	1.4E-04	1.4E-03	1.1E-02	5.4E+03	-5.7E+05	-1.2E+04	2.1E+03
2012	3.7E-03	4.2E-03	2.2E-03	1.4E-04	1.4E-03	1.2E-02	1.1E+04	-5.6E+05	-1.3E+04	2.2E+03
2014	3.8E-03	4.3E-03	2.3E-03	1.4E-04	1.4E-03	1.2E-02	1.8E+04	-5.4E+05	-1.3E+04	2.3E+03

2016	3.9E-03	4.4E-03	2.3E-03	1.5E-04	1.4E-03	1.2E-02	2.4E+04	-5.3E+05	-1.4E+04	2.5E+03
2020	4.2E-03	4.6E-03	2.4E-03	1.5E-04	1.4E-03	1.3E-02	3.6E+04	-5.0E+05	-1.5E+04	2.6E+03
2025	4.5E-03	4.9E-03	2.5E-03	1.6E-04	1.4E-03	1.3E-02	5.1E+04	-4.7E+05	-1.6E+04	3.0E+03
2030	4.8E-03	5.2E-03	2.6E-03	1.7E-04	1.4E-03	1.4E-02	6.6E+04	-4.3E+05	-1.7E+04	3.2E+03
2035	5.1E-03	5.5E-03	2.6E-03	1.8E-04	1.3E-03	1.5E-02	8.2E+04	-4.0E+05	-1.9E+04	3.5E+03
2040	5.4E-03	5.8E-03	2.7E-03	1.9E-04	1.3E-03	1.5E-02	9.7E+04	-3.6E+05	-2.0E+04	3.8E+03
2045	5.7E-03	6.0E-03	2.8E-03	2.0E-04	1.3E-03	1.6E-02	1.1E+05	-3.3E+05	-2.1E+04	4.2E+03
2050	6.0E-03	6.3E-03	2.9E-03	2.1E-04	1.3E-03	1.7E-02	1.3E+05	-2.9E+05	-2.2E+04	4.4E+03

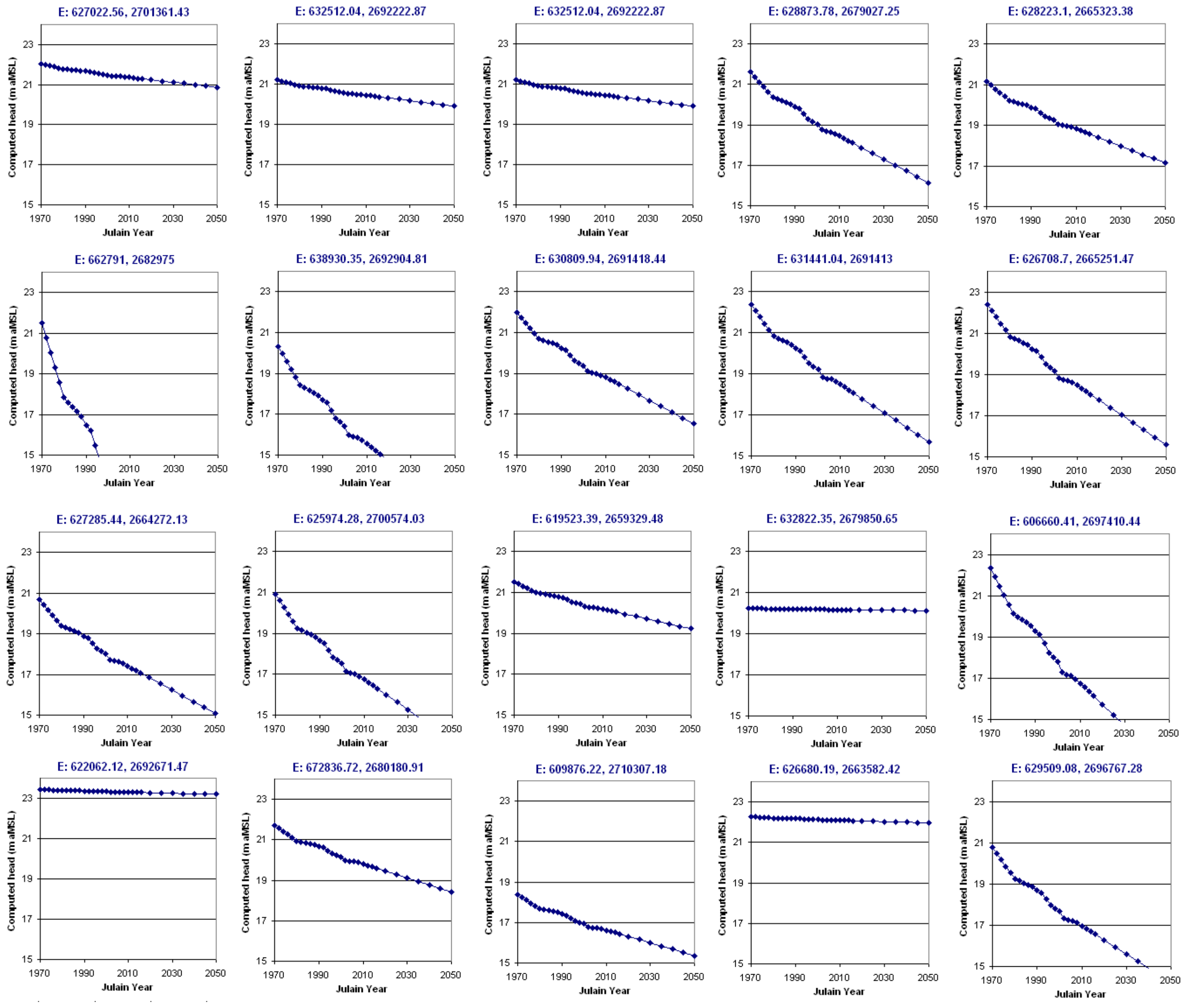

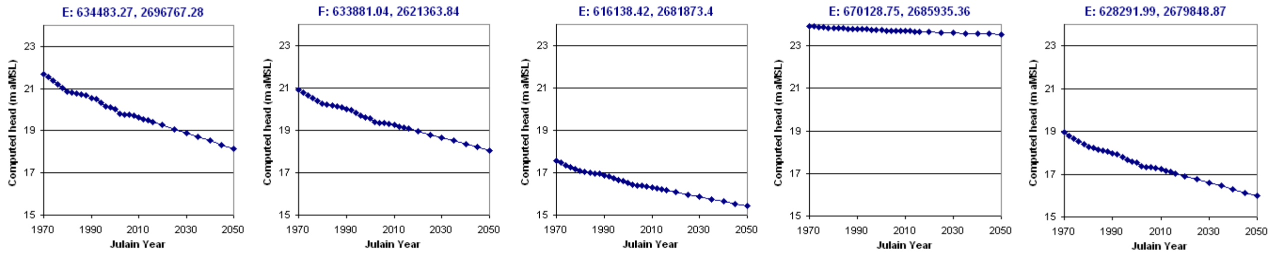

Fig. S6a: Representative computed response curves of hydraulic heads in the Reach E (locations in UTM 45)

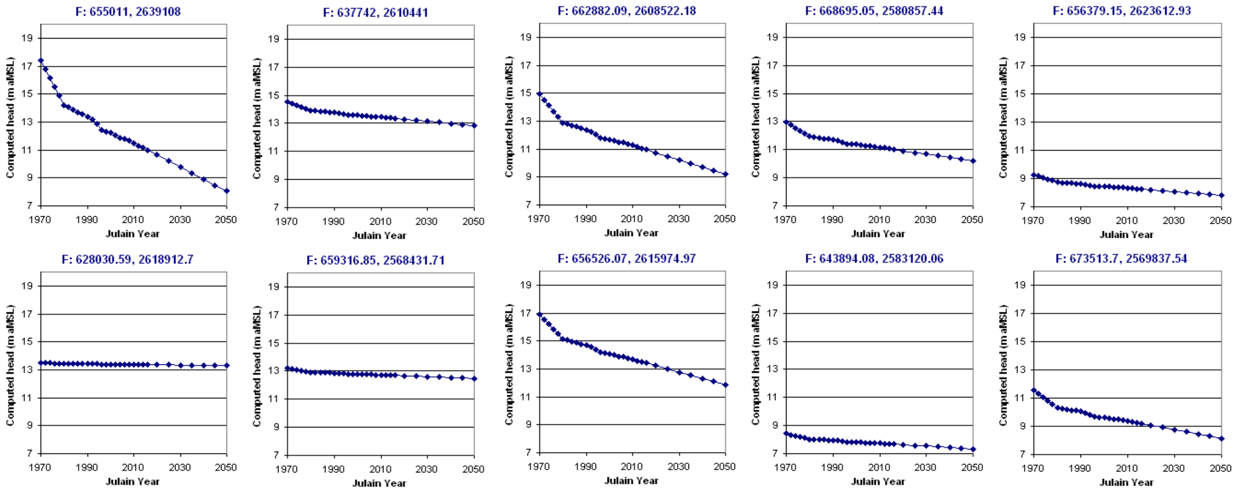

Fig. S6b: Representative computed response curves of hydraulic heads in the Reach G (locations in UTM 45)

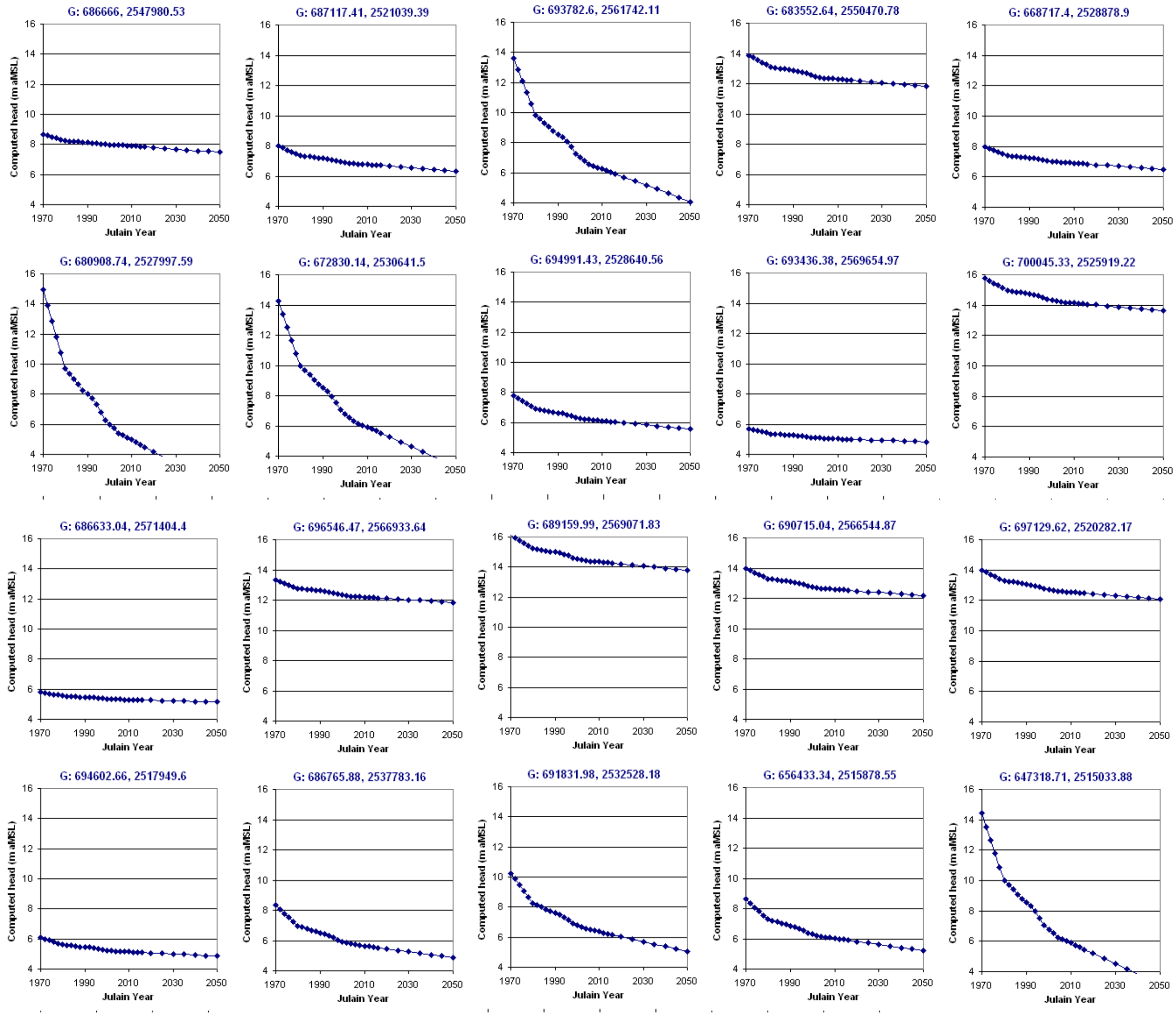

Fig. S6c: Representative computed response curves of hydraulic heads in the Reach G (locations in UTM 45)

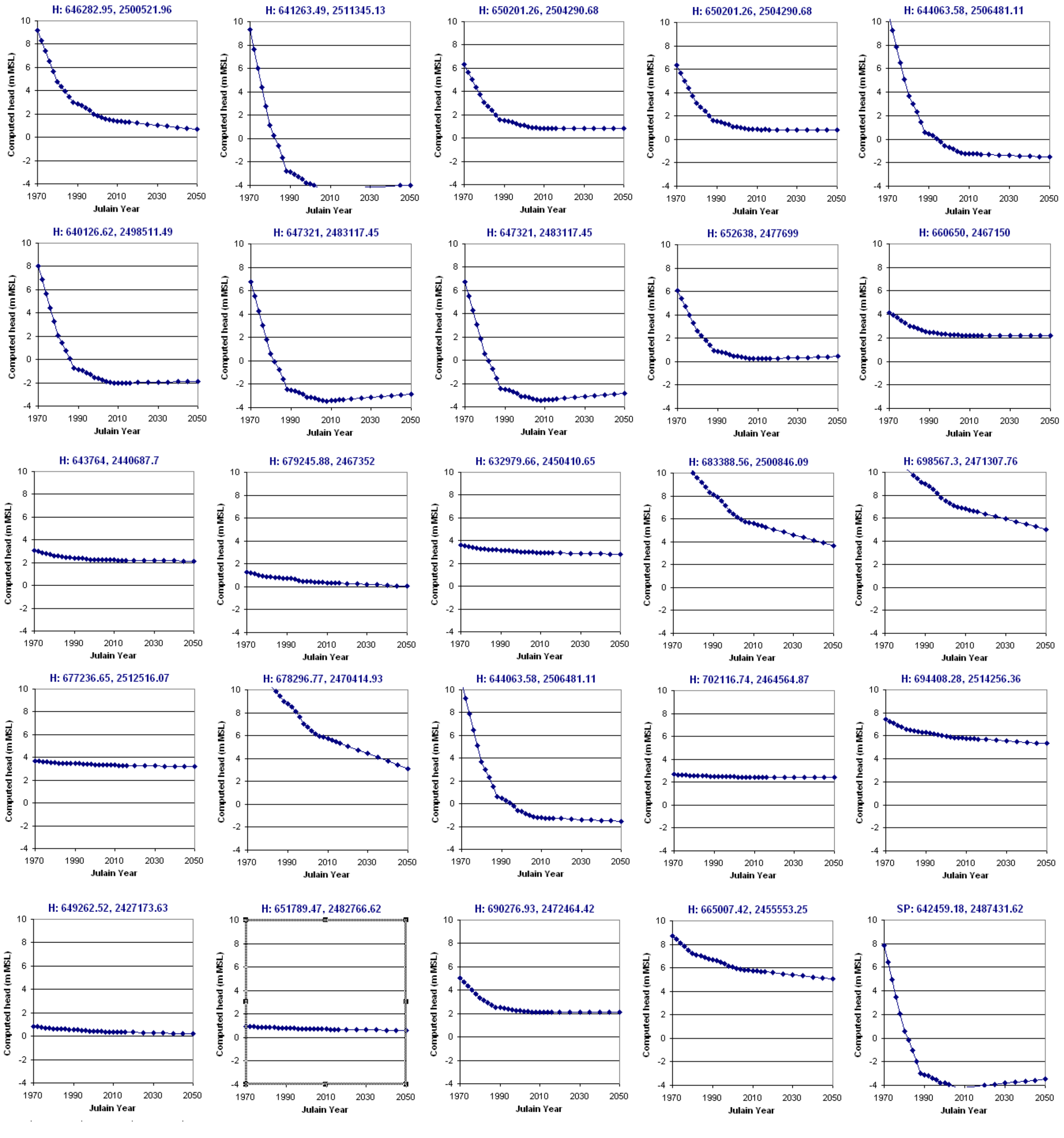

Fig. S6d: Representative computed response curves of hydraulic heads in the Reach H (locations in UTM 45)

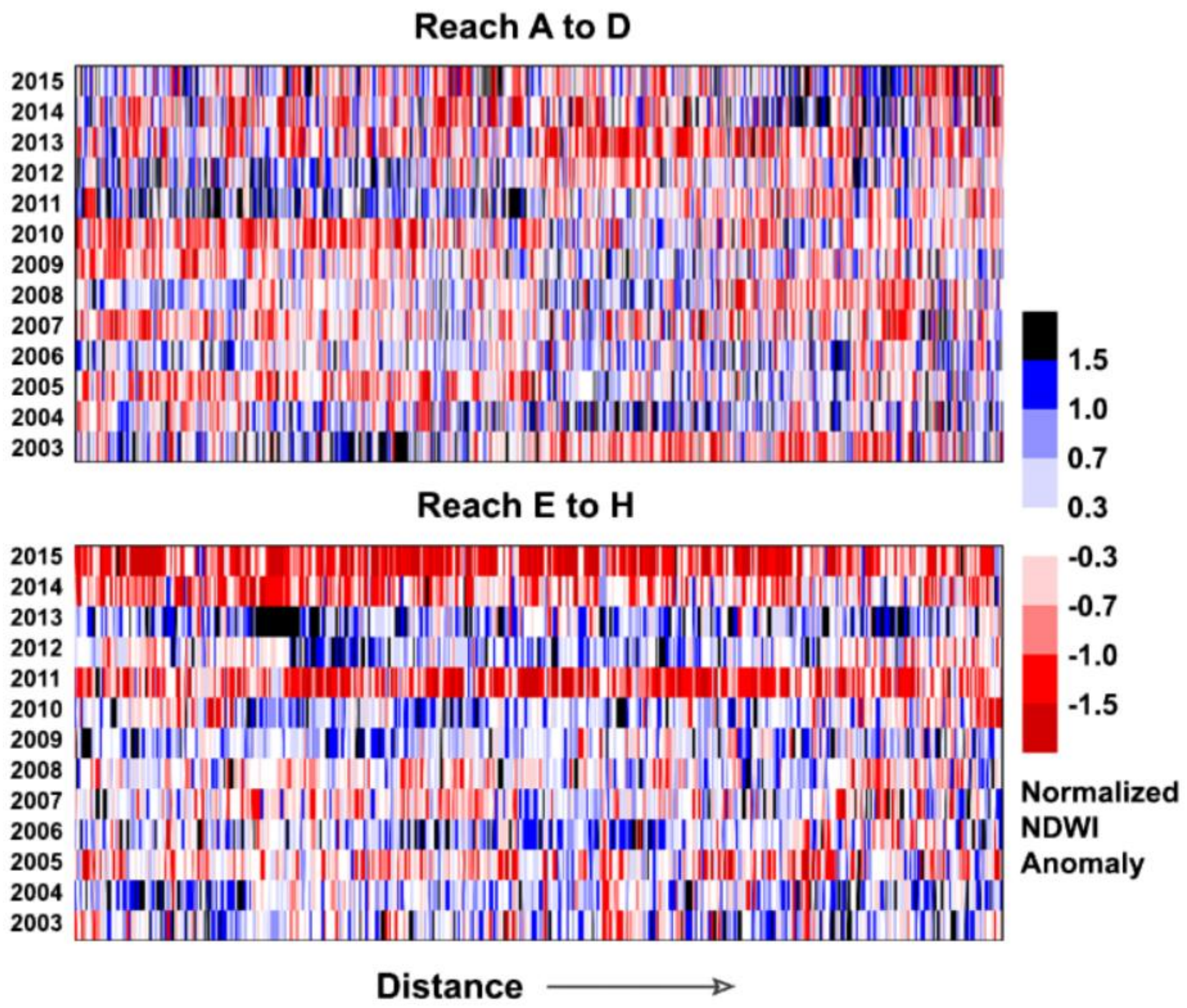

Fig. S7a: Annual mean normalized NDWI anomalies along the Ganges channel

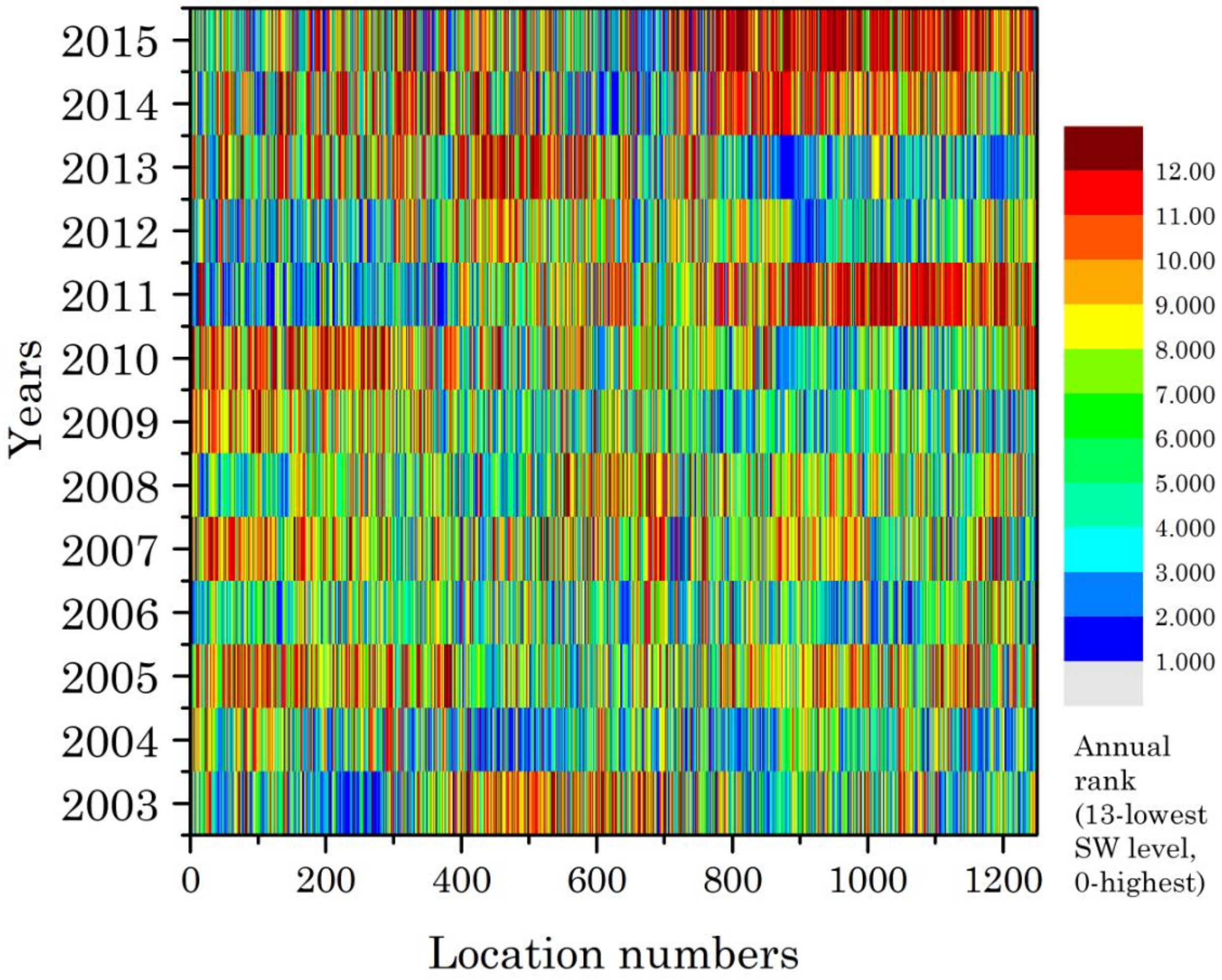

Fig. S7b: Annual ranks of NDWI anomalies along the Ganges channel. The color-scale indicates the relative rank of each year between 2003 and 2015. For example, the value 13 indicates lowest NDWI anomaly observed in the particular year on comparing all of the years

S5 Isotope and hydrogeochemical modeling

In order to find out the percentage contribution of groundwater in Ganges river water, a three component isotope mixing model has been used to characterize the fractions of glacial/ice including snow melt (ICE), surface runoff (SR) and the groundwater discharge/baseflow (GW) component. Isotopic composition ($\delta^{18}O$) and electrical conductivity (EC) values for ice/snow-melt water in higher reaches of Ganges river are collected from Lambs (2000). The relative contribution between glacier and snow melts are not considered separately due to comparatively lesser contribution of both of the components in middle to lower reaches, we used the average value. Isotopic composition and EC values of SR are measured from the samples collected from the major tributaries during pre-monsoon time. Samples are collected from the sites located just before the confluence zone in the tributary. In this process, the SR component would also contain the ICE fraction particularly in Yamuna, Ghagar, Kosi, Fulahar rivers. However, in consideration of conservation of mass, we believe this is the best way for estimating the GW fraction. The $\delta^{18}O$ and EC values from groundwater samples are collected from 227 locations in the periphery of the Ganges river. The unavailability of river water samples corresponding to all of the

groundwater sampling locations led us to use the nearest available river water isotope data of the groundwater sample locations. There are two major assumptions:

1. There are no other components available except those three components, ICE, SR and GW. Total mass of the river water (T) can be represented using mass of the individual components:

$$\text{ICE} + \text{SR} + \text{GW} = \text{T} \qquad (15)$$

2. The average values of $\delta^{18}O$ and EC are not changing in the entire season.

The following equations are solved to calculate the fractions[15].

The sum of fractions of the three components is equal to 1 or 100%.

$$\text{ice} + \text{sr} + \text{gw} = 1 \qquad (16)$$

$$\text{ice} .\delta_{ICE} + \text{sr} .\delta_{SR} + \text{gw} .\delta_{GW} = \delta_T \qquad (17)$$

$$\text{ice} . E_{ICE} + \text{sr} . E_{SR} + \text{gw} . E_{GW} = E_T \qquad (18)$$

Solving these three equations provide the individual fractions, shown below:

$$\text{sr} = \frac{(\delta_T - \delta_{ICE})(E_{GW} - E_{ICE}) - (\delta_{GW} - \delta_{ICE})(E_T - E_{ICE})}{(\delta_{SR} - \delta_{ICE})(E_{GW} - E_{ICE}) - (\delta_{GW} - \delta_{ICE})(E_{SR} - E_{ICE})} \qquad (19)$$

$$\text{gw} = \frac{(\delta_T - \delta_{ICE})(E_{SR} - E_{ICE}) - (\delta_{SR} - \delta_{ICE})(E_T - E_{ICE})}{(\delta_{GW} - \delta_{ICE})(E_{SR} - E_{ICE}) - (\delta_{SR} - \delta_{ICE})(E_{GW} - E_{ICE})} \qquad (20)$$

The main objective of the modeling exercise was to characterize the geochemical pathways and processes that accounts for the chemical changes leading to hydrogeochemical evolution of the shallow groundwater because of mixing with inflowing Ganges river water reaches from the vicinity. Ion activities and saturation indices ($SI = \log(IAP\ K_T^{-1})$, where IAP is the ion activity product and K_T is the equilibrium solubility constant of a phase at ambient temperature) were calculated using PHREEQC (Parkhurst and Appelo, 1999) version 2.17.5.4799 (release date 2010-10-07; with thermodynamic values of phases from the MINTEQ database (minteq.dat, release 3568, date 2009-07-13, Parkhurst, D.L.).

Models for probable, general geochemical mass-transfer reactions along potential groundwater flow paths in the Gangetic aquifers have been developed in this study. The models were generated for our testing the major hypothesis the Ganges river water is inflowing in the aquifers adjoining reaches that area previously known to be gaining. In inverse modeling, the groundwater flow system and chemical composition of the aquifer is assumed to be steady-state conditions. The approach also assumes that the initial and final end member waters that are used in the simulations are representative groundwaters from the same, or convergent flow path(s), and accordingly the minerals employed in the model calculations must occur within the aquifer matrix. Further, it is presumed that the effects of hydrodynamic dispersion along the modeled flow paths are negligible (Plummer et al., 1983; Zhu and Anderson, 2002; Johannesson and Tang, 2009). Ion-exchange reactions (between CaX_2, MgX_2, NaX and KX) are modeled following the Gaines-Thomas convention (Gaines and Thomas, 1953) and equilibrium constants (Appelo and Postma, 1993; Fryar et al., 2001; Mukherjee and Fryar, 2008):

$$1/iI^{i+} + 1/jJ{-}X_j = 1/iI{-}X_i + 1/jJ^{j+} \qquad (21)$$

where X represents the solid phase exchanger. Following procedures of Fryar *et al.*(2001), Mukherjee and Fryar (2008) and Raychowdhury *et al.*(2014), minimal reaction-path (inverse) models were developed using PHREEQC (Parkhurst and Appelo, 1999) version 2.17 for pairs of wells located along possible flowpaths, as suggested by previous studies (Mukherjee et al., 2007; Mukherjee and Fryar, 2008; Mukherjee et al., 2012). The selected pairs of wells used as end members are only based on their distinctiveness in hydrochemistry, and they are located up gradient-down gradient to each other along suggested general flow direction. The inaccuracy in the choices of end-member waters and in analytical data was handled by including uncertainties (≤25%, incrementally increasing from an initial 5% default) in the inverse modeling. The importance of this exercise is for testing of hypothesis for possible, "general" type of groundwater-surface water reactions, regionally, within the study areas and not specific to any particular flow path or pairs/sets of wells. A total of 76 different inverse geochemical models in the eight aquifers were executed within the acceptable uncertainty range, resulting to 1377 solutions. All of the modeled reaction flow paths were designed for evolution through silicate weathering (quartz, amorphous silica, feldspars, clay minerals etc.), cation exchange (Ca^{2+}/Mg^{2+},Ca^{2+}/Na^{+}, Ca^{2+}/K^{+}, Na^{+}/Mg^{2+}, and Na^{+}/K^{+}) and carbon cycling (CO_2 dissolution and exsolution; CH_2O (organic carbon) oxidation) or combination of these along each flow path in order to explain the gross hydrochemistry. The major hydrogeochemical reactions that were considered in the models include:

In natural systems, carbonate mineral dissolution can be written as (Garrels and Mackenzie, 1971):

$CaCO_3$ (calcite) + $H_2CO_3 \rightarrow Ca^{2+} + 2HCO_3^-$ (22a)

$CaMg(CO_3)_2$ (dolomite) + $H_2CO_3 \rightarrow Ca^{2+} + Mg^{2+} + 4HCO_3^-$ (22b)

$CaCO_3 + H_2SO_4 \rightarrow Ca^{2+} + SO_4^- + H_2CO_3$ (23a)

$CaMg(CO_3)_2 + 2H_2SO_4 \rightarrow Ca^{2+} + Mg^{2+} + 2SO_4^- + 2H_2CO_3$ (23b)

Reactions involving silicate weathering by incongruent dissolution can be generalized by following Appelo and Postma (1994), Drever (1997) and Faure (1998) as:

$2KAlSi_3O_8$ (orthoclase) + $2CO_2 + 3H_2O \rightarrow$ clay minerals (e.g. kaolinite ($Al_2Si_2O_5(OH)_4$)) + $2K^+ + 2HCO_3^- + 4SiO_2$ (24a)

$2NaAlSi_3O_8$ (plagioclase (albite)) + $2CO_2 + 3H_2O \rightarrow$ clay minerals (e.g. kaolinite) + $2Na^+ + 2HCO_3^- + 4SiO_2$ (24b)

$CaAlSi_2O_8$ (plagioclase (anorthite)) + $2CO_2 + 3H_2O \rightarrow$ clay minerals (e.g. kaolinite) + $Ca^{2+} + 2HCO_3^-$ (24c)

$2K(Mg_2Fe^{2+})AlSi_3O_{10}(OH)_2$ (biotite) + $10CO_2 + 0.5O_2 + 8H_2O \rightarrow$ 2 clay minerals (e.g. kaolinite) + $2Fe(OH)_3 + 2K^+ + 4Mg^{2+} + 10HCO_3^- + 4SiO_2$ (25a)

$2CaMgFeAl_2Si_3O_{12}$ (pyroxene) + $8CO_2 + 0.5O_2 + 11H_2O \rightarrow$ 2 clay minerals (e.g. kaolinite) + $2Fe(OH)_3 + 2Ca^{2+} + 2Mg^{2+} + 8HCO_3^- + 2SiO_2$ (25b)

The reaction for cation exchange can be generalized as (following Karanth, 1994):

$0.5Ca^{2+}$ + Na-clay $\rightarrow$ 0.5Ca-clay + Na^+ (25c)

The models were balanced on Cl^- concentrations (assuming Cl^- as a conservative solute), and halite dissolution was used for 23 models as a proxy for mixing with connate water from the proto-Bay of Bengal. Because of uncertainty in the measurements of Al (not detected at most locations), and the fact that inclusion of alumino-silicate phases led to an impractically large number of preliminary model solutions, Al was not used in the final reaction-path models. Instead, Silica balance was used to evaluate the role of silicate weathering, as a Si-balanced model would indicate minimal Si influx from weathering, whereas a failure would suggest substantial silicate hydrolysis along the flow path.

S6 Non-parametric statistical analysis

The Hodrick-Prescott (HP) (Hodrick and Prescott, 1997) filter, a non-parametric trend estimator, has been used in this study for computing the trend analysis. The trends and cycles are separated in this approach upon solving the following equation (Hodrick and Prescott, 1997):

$$\text{Min (T)} \sum_{t=1}^{T}((y_t - T_t)^2 + \lambda((T_{t+1} - T_t) - (T_t - T_{t-1}))^2 \quad (26)$$

Where T_{t+1} and T_{t-1} are the trend component at the time steps *t+1* and *t-1*, respectively. The cyclical components can be obtained after removing the trends from its real values; also, the long-term mean of the cyclical component closes to zero (Hodrick and Prescott, 1997). Variability in cyclical components are reduced through the selection of a suitable value for the smoothing parameter (λ) (Hodrick and Prescott, 1997). The selected value of λ for quarterly data is 1600 (Hodrick and Prescott, 1997; Ravn and Uhlig, 2002). The trend values are estimated following Sen's slope method (Sen, 1968). The non-parametric Sen's slope approach calculates the regression co-efficient using Kendal's rank correlation estimates (Sen, 1968).

S7 Food security estimates

Total cereal production during Rabi season (cultivation during pre-monsoon time, cropping time October-March and growing time March to June) data are collected state-wise (Uttar Pradesh, Bihar, Jharkhand and West Bengal) during 2010 to 2013 (source: https://data.gov.in/, accessed on January 15, 2016). Total cereal productions during Rabi (winter) season are 41.2 to 44.1 million tonnes in the study area. Total cereal production during Kharif (monsoon) season (cultivation during monsoon time, cropping time July-October) has also been used to get the total annual cereal production (source: https://data.gov.in/). The baseflow component is reaching negative values or river capture condition in 2050 in most of the lower reaches with an average value ~0 (Fig. 3). Therefore the surface water quantity will be reduced as a function of near zero contribution from baseflow component in 2050. This will lead to reduction in surface water irrigation during pre-monsoon time. Assuming no change in total precipitation pattern, groundwater irrigation pattern, cropping area, crop type, crop yield and crop import and export, we have predicted the amount of total cereal production during pre-monsoon/Rabi season. Average daily energy requirement for an adult is assumed to be 2500 cal/gram (FAO, 2004), with carbohydrate contribution approximately 50%. Total dietary energy requirement from carbohydrates, has been estimated for the projected population in 2050. Total cereal production in 2050 has been estimated using a combination of the reduced total cereal during Rabi season and the unchanged total cereal production for Kharif season. Crop deficiency values are estimated for 2050 and reported as number of people affected. The flow-chart (Fig. S8) showing the steps followed for estimating the number of population effected.

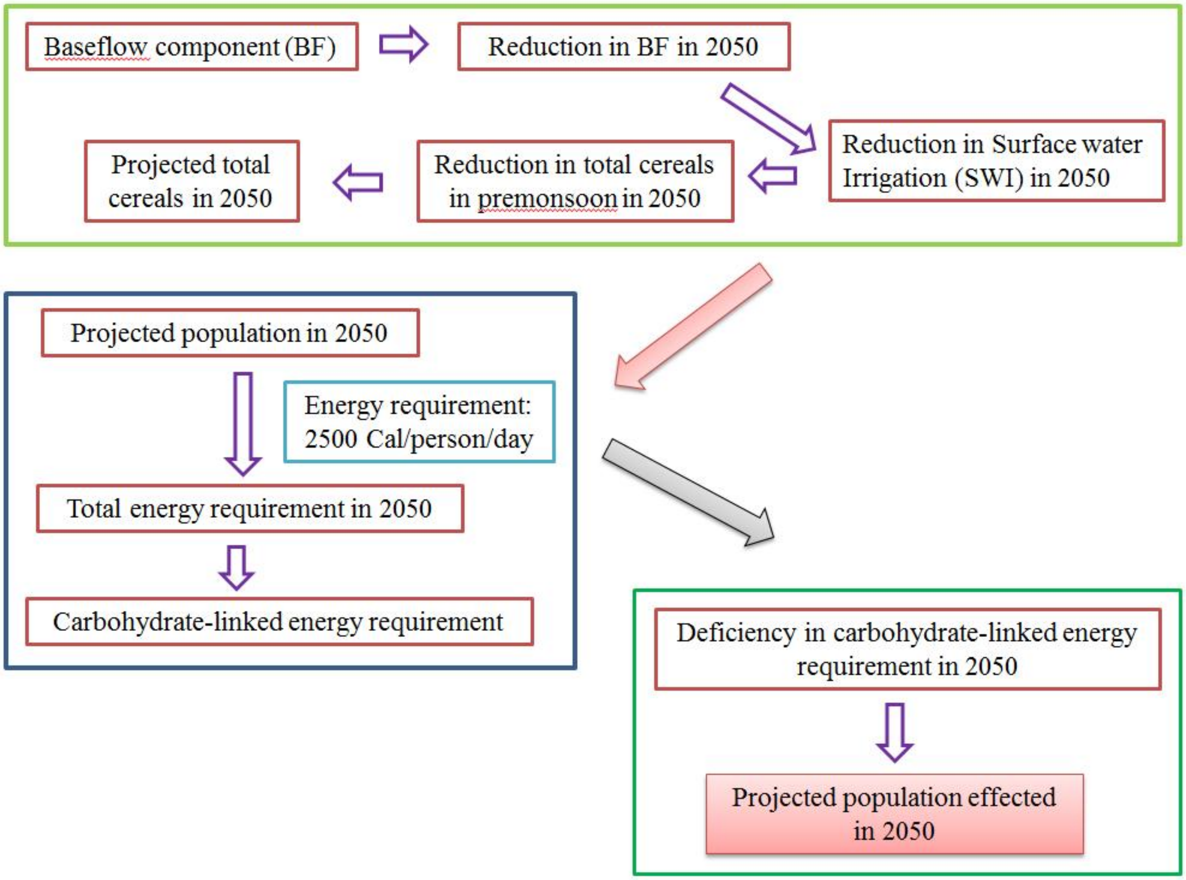

Fig. S8: Flow-chart of steps taken for estimating food security issues

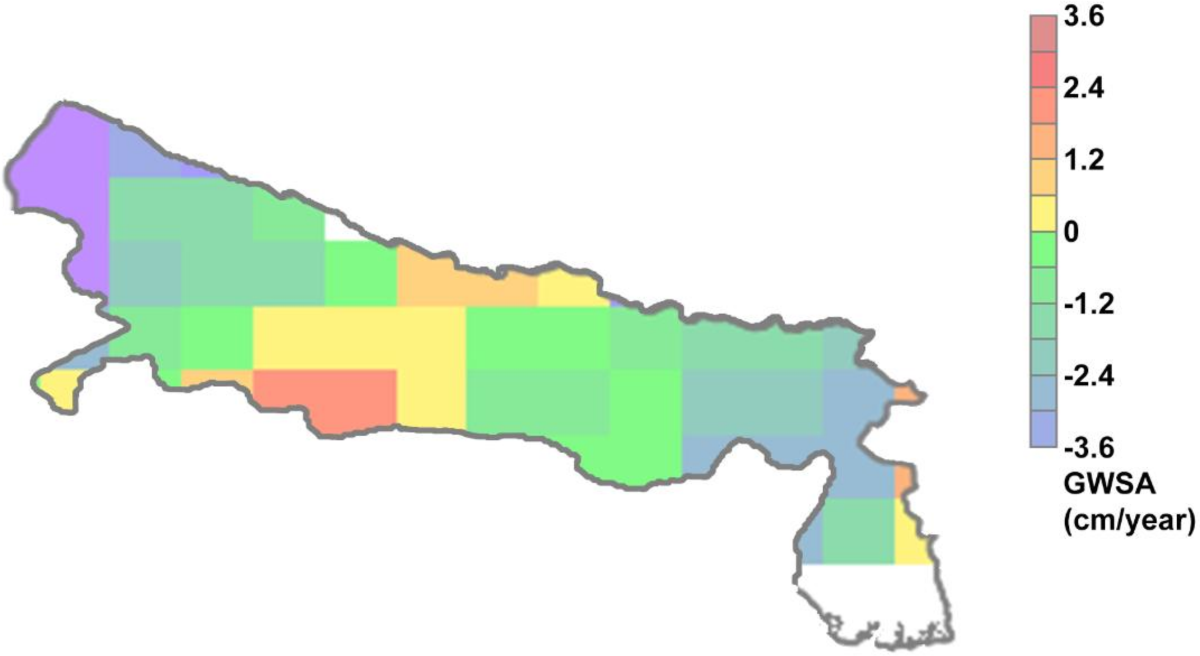

Fig. S9: Trends in satellite-based, gridded ($1^0 \times 1^0$) GWS anomalies in 2003-2014. All the maps were made using Ferret program (NOAA) and standard graphical illustrators

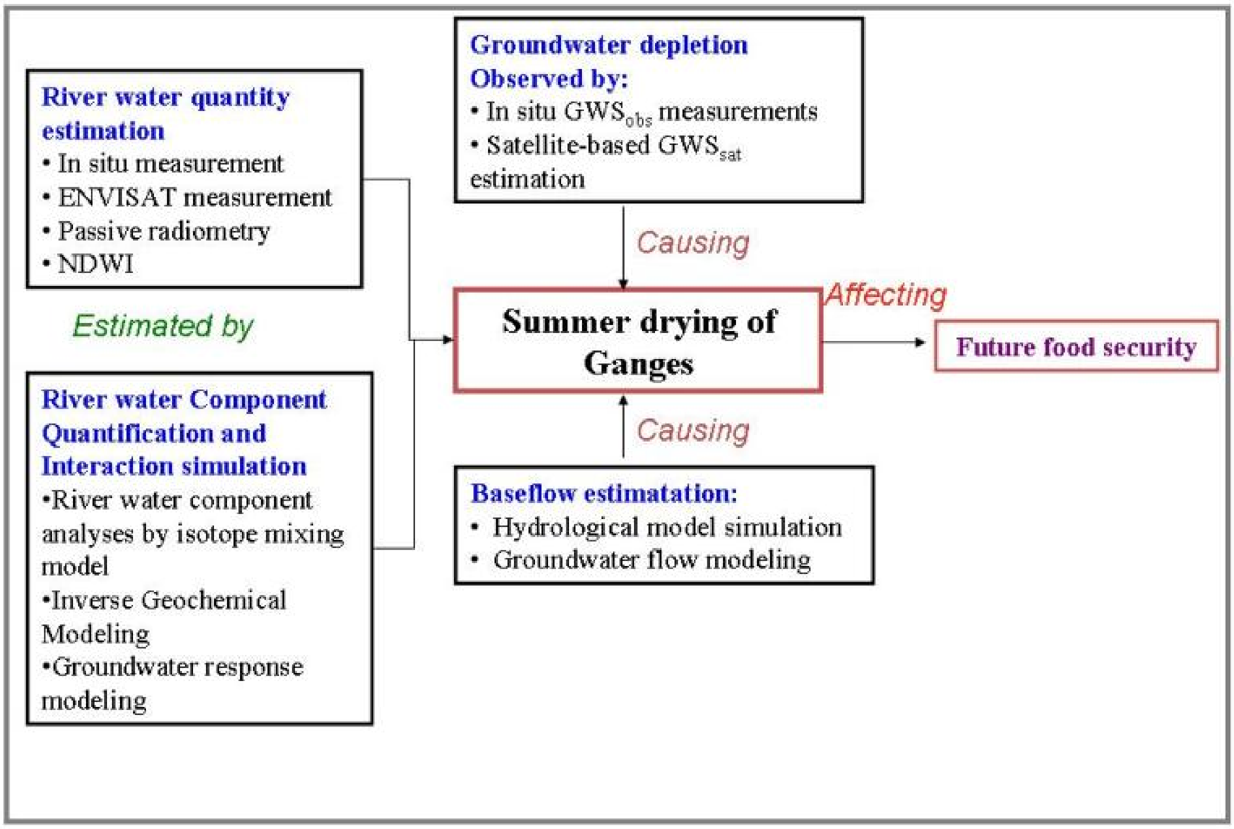

Fig. S10: Summary of methods used for this study

Appelo, C. A. J. & Postma, D. Geochemistry, Groundwater and Pollution. Balkema (1993).

Appelo, C. A. J. Geochemistry Groundwater and Pollution (1994).

Bhanja, S. N. *et al.* Validation of GRACE based groundwater storage anomaly using in situ groundwater level measurements in India. *Journal of Hydrology* **543**, 729-738 (2016).

Cheng, M., Ries, J. C., & Tapley, B. D. Variations of the Earth's figure axis from satellite laser ranging and GRACE. *Journal of Geophysical Research: Solid Earth* **116** (2011).

Drever, J. I. The Geochemistry of Natural Waters (3rd ed.). Prentice-Hall, Upper Saddle River, NJ (1997).

Dürr, H. H., Meybeck, M., & Dürr, S. H. Lithologic composition of the Earth's continental surfaces derived from a new digital map emphasizing riverine material transfer. *Global Biogeochemical Cycles* ***19*** (2005).

FAO-Human energy requirements Report of a Joint FAO/WHO/UNU Expert Consultation Rome, 17-24 October 2001. FAO Food and nutrition technical report series 1 (FAO, Rome, 2004).

Faure, G. Principles and Applications of Geochemistry (2nd ed.). Prentice Hall, Upper Saddle River, NJ (1998).

Fryar, A. E., Mullican, W. F., & Macko, S. A. Groundwater recharge and chemical evolution in the southern High Plains of Texas, USA. *Hydrogeol. J.* **9**, 522-542 (2001).

Gaines Jr, G. L. & Thomas, H. C. Adsorption studies on clay minerals. II. A formulation of the thermodynamics of exchange adsorption. *The Journal of Chemical Physics* **21**, 714-718 (1953).

Garrels, R. M. & Mackenzie, F. T. Evolution of sedimentary rocks (1971).

Geruo, A., Wahr, J., & Zhong, S. Computations of the viscoelastic response of a 3-D compressible Earth to surface loading: an application to Glacial Isostatic Adjustment in Antarctica and Canada. *Geophysical Journal International* **192**, 557-572 (2013).

Hodrick, R. J. & Prescott, E. C. Postwar US business cycles: an empirical investigation. *Journal of Money, credit, and Banking* 1-16 (1997).

Johannesson, K. H. & Tang, J. Conservative behavior of arsenic and other oxyanion-forming trace elements in an oxic groundwater flow system. *Journal of Hydrology* **378**, 13-28 (2009).

Karanth, K. R. Groundwater assessment development and management. Tata McGraw-Hill Publishing Company Limited, New Delhi, Third reprint (1994).

Lambs, L. Correlation of conductivity and stable isotope 18O for the assessment of water origin in river system. *Chemical Geology* **164**, 161–170 (2000).

Mace, R. E. Estimating transmissivity using specific-capacity data. Geological Circular 0102, Bureau of Economic Geology, University of Texas at Austin (2001).

Ministry of Water Resources (MoWR). First Minor Irrigation Census, 1986-87. Minor Irrigation Division, G.o.I., New Delhi (1993).

Ministry of Water Resources (MoWR). Second Minor Irrigation Census, 1993-94. Minor Irrigation Division, G.o.I. , New Delhi (2001).

Ministry of Water Resources (MoWR). Third Minor Irrigation Census, 2000-2001. Minor Irrigation Division, G.o.I. , New Delhi (2005).

Ministry of Water Resources (MoWR). Fourth Minor Irrigation Census, 2006-2007. Minor Irrigation Division, G.o.I. , New Delhi (2013).

Mukherjee, A. & Fryar, A. E. Deeper groundwater chemistry and geochemical modeling of the arsenic affected western Bengal basin, West Bengal, India. *Applied Geochemistry* **23**, 863-894 (2008).

Mukherjee, A. *et al*. Hydrogeochemical comparison and effects of overlapping redox zones on groundwater arsenic near the Western (Bhagirathi sub-basin, India) and Eastern (Meghna sub-basin, Bangladesh) margins of the Bengal Basin. *Journal of Contaminant Hydrology* **99**, 31-48 (2008).

Mukherjee, A. *et al*. Solute chemistry and arsenic fate in aquifers between the Himalayan foothills and Indian craton (including central Gangetic plain): Influence of geology and geomorphology. *Geochimica et Cosmochimica Acta* **90**, 283-302 (2012).

Parkhurst, D. L. & Appelo, C. A. J. User's guide to PHREEQC (version 2): a computer program for speciation, batch-reaction, one-dimensional transport, and inverse geochemical calculations. US Geol. Surv. Water-Resour. Invest. Rep. 99-4259 (1999).

Plummer, L. N., Parkhurst, D. L., & Thorstenson, D. C. Development of reaction models for ground-water systems. *Geochimica et Cosmochimica Acta* **47**, 665-685 (1983).

Ravn, M. O. & Uhlig, H. On adjusting the Hodrick-Prescott filter for the frequency of observations. *Review of economics and statistics* **84**, 371-376 (2002).

Raychowdhury, N. *et al*. Provenance and fate of arsenic and other solutes in the Chaco-Pampean Plain of the Andean foreland, Argentina: From perspectives of hydrogeochemical modeling and regional tectonic setting. *Journal of Hydrology* **518**, 300-316 (2014).

Sutanudjaja, E. H., Van Beek, L. P. H., De Jong, S. M., van Geer, F. C., & Bierkens, M. F. P. Large-scale groundwater modeling using global datasets: a test case for the Rhine-Meuse basin. *Hydrology and Earth System Sciences* ***15,*** 2913 (2011).

Swenson, S. C., Chambers, D. P., & Wahr, J. Estimating geocenter variations from a combination of GRACE and ocean model output. *J. Geophys. Res.-Solid Earth* **113**, B08410 (2008).

Zhu, C. & Anderson, G. Environmental applications of geochemical modeling. Cambridge University Press (2002).

Wada, Y., Wisser, D., & Bierkens, M. F. P. Global modeling of withdrawal, allocation and consumptive use of surface water and groundwater resources. *Earth System Dynamics* ***5***, 15 (2014).

Wada, Y., de Graaf, I. E., & van Beek, L. P. High-resolution modeling of human and climate impacts on global water resources. *Journal of Advances in Modeling Earth Systems* **8**, 735-763 (2016).

Watkins, M. M. *et al.* Improved methods for observing Earth's time variable mass distribution with GRACE using spherical cap mascons. *J. Geophys. Res. Solid Earth* **120** (2015).
